# GerAB residues predicted to interact with water based on MD simulations mediate germinosome stability in *Bacillus subtilis* spores

**DOI:** 10.3389/fmicb.2026.1800262

**Published:** 2026-04-29

**Authors:** Longjiao Chen, Silke van Buuren, George Korza, Jocelyne Vreede, Peter Setlow, Stanley Brul

**Affiliations:** 1 Swammerdam Institute for Life Sciences, University of Amsterdam, Amsterdam, Netherlands; 2 Van’t Hoff Institute for Molecular Sciences, University of Amsterdam, Amsterdam, Netherlands; 3 Department of Molecular Biology and Biophysics, UConn Health, Farmington, CT, United States

**Keywords:** APC transporters, bacterial spores, germinant receptor, MD simulations, spore germination

## Abstract

Some species in the *Bacillales* and *Clostridiales* orders form spores under unfavourable environmental conditions. These spores are metabolically dormant and highly resistant to extreme stress. The spore core—analogous to the protoplast of vegetative cells—contains only 25–45% water by wet weight, compared to ~80% in vegetative cells. Upon activation by small-molecule nutrients, spores germinate, restoring their core water content, restoring metabolism and becoming easy to kill and then progressing through outgrowth to vegetative growth. GerAB is the B subunit of the prototypical *Bacillus subtilis* GerA germinant receptor (GR), a membrane protein belonging to the Amino Acid–Polyamine–Organocation (APC) superfamily of transporters. It functions as the L-alanine sensor that initiates germination and was previously predicted, based on molecular dynamics (MD) simulations, to contain a putative water channel. Using MD simulations, we identified low amount of water permeating through GerAB (ranging from 1 to 121 water molecule/μs in 10 parallel MD simulations), thus revealing a water pathway in GerAB that diverges from the L-alanine binding pocket, suggesting that water transport may play roles in germination beyond facilitating ligand binding. Analysis of water–residue contact frequencies identified eight hydrophilic residues lining the water’s path. Individual substitution of high-contact residues with similarly sized non-polar residues impaired L-alanine germination and disrupted GerAB structural integrity as assessed by Western Blotting. These mutants also respond to the AGFK germinant mixture (L-asparagine, D-glucose, D-fructose and potassium) in slower, yet individually distinct kinetics compared to that of wild-type (wt) spores. These findings prove that water contact residues in GerAB predicted by MD simulations are crucial for the stability of this protein and thus the germinosome complex with all GRs.

## 1 Introduction

Certain species within the *Bacillales* and *Clostridiales* orders can form dormant spores in response to harsh environmental conditions, especially growth-limiting physiological conditions like changes in pH or temperature, the presence of reactive oxygen species, and the lack of nutrients. These spores are metabolically inactive and exhibit exceptional resistance to a wide range of extreme treatments, such as high temperatures, desiccation, radiation, and a variety of chemical reagents including antibiotics. However, potentially, spores from species such as *Bacillus cereus*, *Bacillus anthracis*, and *Clostridioides difficile* can cause food spoilage, and foodborne illnesses (Christie and Setlow, 2020). Consequently, the mechanisms underlying spore germination in these bacteria have been investigated over the past decades, especially so since spores lose their extreme resistance properties upon germination and are easy to kill. A characteristic feature of bacterial spores is their low core water content. In *Bacillus subtilis*, the spore core—analogous to the cytoplasm of vegetative cells—contains only 25–45% water by wet weight, compared to approximately 80% in vegetative cells. This low water capacity in the spore core contributes to the stabilization of core DNA, the low metabolism of dormant spores and spores’ resistance to environmental stress. Upon activation by small-molecule nutrients known as germinants, spores initiate germination, during which rapid water uptake occurs. This water influx leads to roughly a two-fold expansion of the spore core volume, a process that takes around 10 min in an individual spore (Setlow et al., 2016; Camilleri et al., 2019; Christie and Setlow, 2020). Full hydration of the core marks the completion of germination and enables the resumption of metabolic activity and macromolecular synthesis. Despite its importance, the molecular mechanism by which water is taken up into the spore core during germination remains unknown in spore-forming bacteria (Christie and Setlow, 2020).

During the germination process described above, germinant receptors (GRs) play a central role by sensing germinants and initiating the molecular cascade that leads to spore revival. There are three functional GRs in the model organism *Bacillus subtilis*, GerA, GerB and GerK, with GerA responding to L-alanine while GerB and GerK respond to a mixture of AGFK (L-asparagine, D-glucose, D-fructose and potassium) in a cooperative manner (Christie and Setlow, 2020). Each GR is composed of three essential subunits, and it has been well-established that all three subunits are indispensable for forming a functional receptor (Griffiths et al., 2011). The three GRs further colocalize with other germination proteins in a protein complex termed a germinosome, in which each component is structurally and functionally dependent on each other (Griffiths et al., 2011; Wen et al., 2019). Recent studies have significantly advanced the understanding of GR function by showing that the three subunits of GerA assemble into a heteropentameric ion channel, where the GerAA subunit forms a pentameric structure that facilitates the release of monovalent cations upon detection of the germinant L-alanine bound to the GerAB subunit (Artzi et al., 2021; Gao et al., 2023). These findings were made possible by a combination of AlphaFold structure predictions and mutagenesis experiments. Beyond the nutrient sensing function of the B subunit, Blinker *et al*. identified a putative water channel within GerAB using molecular dynamics (MD) simulations, suggesting a novel mechanism for water transport during germination. More recently, Chen et al. (2025) further characterized this water channel by identifying specific residues in GerAB that form a blockade for water crossing in steered MD simulations. These studies point to a previously less understood role for GerAB in facilitating core hydration, a vital step in spore germination. However, experimental validation, the presumed regulation of this water channel and its precise physiological role in spore germination remain open questions. Given its possible multifunctionality, GerAB makes a fascinating target for elucidating the molecular mechanisms of spore germination.

As no structure has been determined for GerAB with experimental methods, several studies have sought to deepen the understanding of its function with bioinformatics approaches. These investigations have reached a consensus that GerAB is a member of the membrane transporter protein superfamily known as the Five-Helix Inverted Repeat Superfamily. More specifically, GerAB belongs to the Amino Acid–Polyamine–Organocation (APC) superfamily (Cameron et al., 2013; Artzi et al., 2021; Blinker et al., 2021). Notably, growing evidence points to the critical role of water in the function of proteins within this superfamily. This holds true for two prominent families within the Five-Helix inverted repeat superfamily, the Amino Acid–Polyamine–Organocation (APC) family and the Neurotransmitter–Sodium Symporter (NSS) family. For example, a well-characterized APC transporter, GkApcT from *Geobacillus kaustophilus*, has been shown to transport water molecules *in silico* with MD simulations (Afshinpour et al., 2023). In the absence of quantitative measurements of GkApcT permeability, the study indicates that its water permeability is lower than that of dedicated aquaporins, which exhibit water transport rates on the order of $10^{9}s^{-1}$ in AQP1 (Sansom and Law, 2001). In GkApcT, water facilitates substrate translocation by acting as a solvent medium, stabilizing nearby residues, and supporting conformational changes required for transport (Afshinpour et al., 2023). Similarly, in the NSS family, the sodium–galactose symporter vSGLT functions not only as a transporter of galactose but also mediates water transport, acting simultaneously as a passive water channel and an active transporter in MD simulations (Choe et al., 2010). Importantly, *B. subtilis* GerAB is thought to share a high degree of structural homology with GkApcT (Artzi et al., 2021) and LeuT, another well-studied NSS family transporter (Shaffer et al., 2009; Del Alamo et al., 2022). Additionally, molecular dynamics simulations have identified a putative water channel within GerAB (Blinker et al., 2021), with a pore radius of 0.1–0.3 nm throughout 5 parallel 100 ns simulations at all times. This supports the hypothesis that water may play a functional role in GerAB. Unlike canonical transporters, GerAB and its homologs may act as *transceptors*—proteins that sense external ligands and trigger downstream signalling without necessarily transporting the ligand itself (Taylor, 2009; Moir and Cooper, 2015). Nonetheless, given the critical role water plays in structurally similar transporters, it seemed worthwhile to explore if water transport is also a function of GerAB, thus providing new insight of the role of this GR subunit in spore germination.

To further investigate the mechanism of GerAB’s interaction with water molecules and the role of hydration in spore germination, we performed molecular dynamics simulations of the GerAB–membrane system. These simulations revealed water crossing through GerAB at a low level (ranging from 1 to 121 molecules/μs in 10 parallel MD simulations). Analysing contact frequency between GerAB residues and crossing water molecules identified eight hydrophilic residues that frequently interact with water during crossing events. These residues span the GerAB protein on TM regions 1, 2, 3, 6, and 10, and diverge from the previously revealed L-alanine binding pocket (Artzi et al., 2021). To experimentally verify the functional role of these residues, we substituted each one individually with similarly sized hydrophobic amino acid residues and prepared mutant spores. In germination assays, all of the mutant GerAB spores showed defects in L-alanine induced germination. Furthermore, Western Blot analyses revealed impaired GerAB structural integrity. In addition, AGFK induced germination in GerAB mutant spores, which also displayed decreased and individually varied kinetics compared to wt spores. These results indicated that different mutations in GerAB introduce different structural perturbations that eventually affect the function of GerB and GerK GR in various ways. Together, these results demonstrate that the predicted water contact residues in GerAB are structural hotspots in this protein and likely affect germinosome stability.

## 2 Results

### 2.1 Low amount of water passage occurred during MD simulations

The root mean square deviation (RMSD) of protein carbon alpha (CA) atoms indicated that the structure fluctuation stabilized around 0.6 nm after approximately 200 ns, suggesting the system had reached equilibrium beyond this time point (Supplementary Figure S1A). CA root mean square fluctuation (RMSF) analysis across ten independent simulations showed consistent trends: low fluctuations in the transmembrane helical regions and high fluctuations in loop regions (Supplementary Figure S1B). To compare our GerAB structural model with the AlphaFold prediction, we first compared the structural deviation between AlphaFold prediction and RaptorX prediction represented by per-residue CA deviation (Supplementary Figure S1C). The overall RMSD between the two structures is 0.51 nm (Supplementary Figure S1D), showing an overall consensus of GerAB fold between two models. The CA deviation is lower at TM helical regions (less than 0.3 nm) and higher at loop regions (around 0.5 nm) and both termini (reaching 1–2 nm). This indicates the two models exhibit agreements at the TM regions and less agreement regarding the flexible loops and termini. CA RMSD of each simulation run with the AlphaFold structure as a reference stabilizes around 0.5 nm (Supplementary Figure S1E), indicating that our current GerAB model maintains its consensus with AlphaFold prediction during simulations.

**Figure 1:**
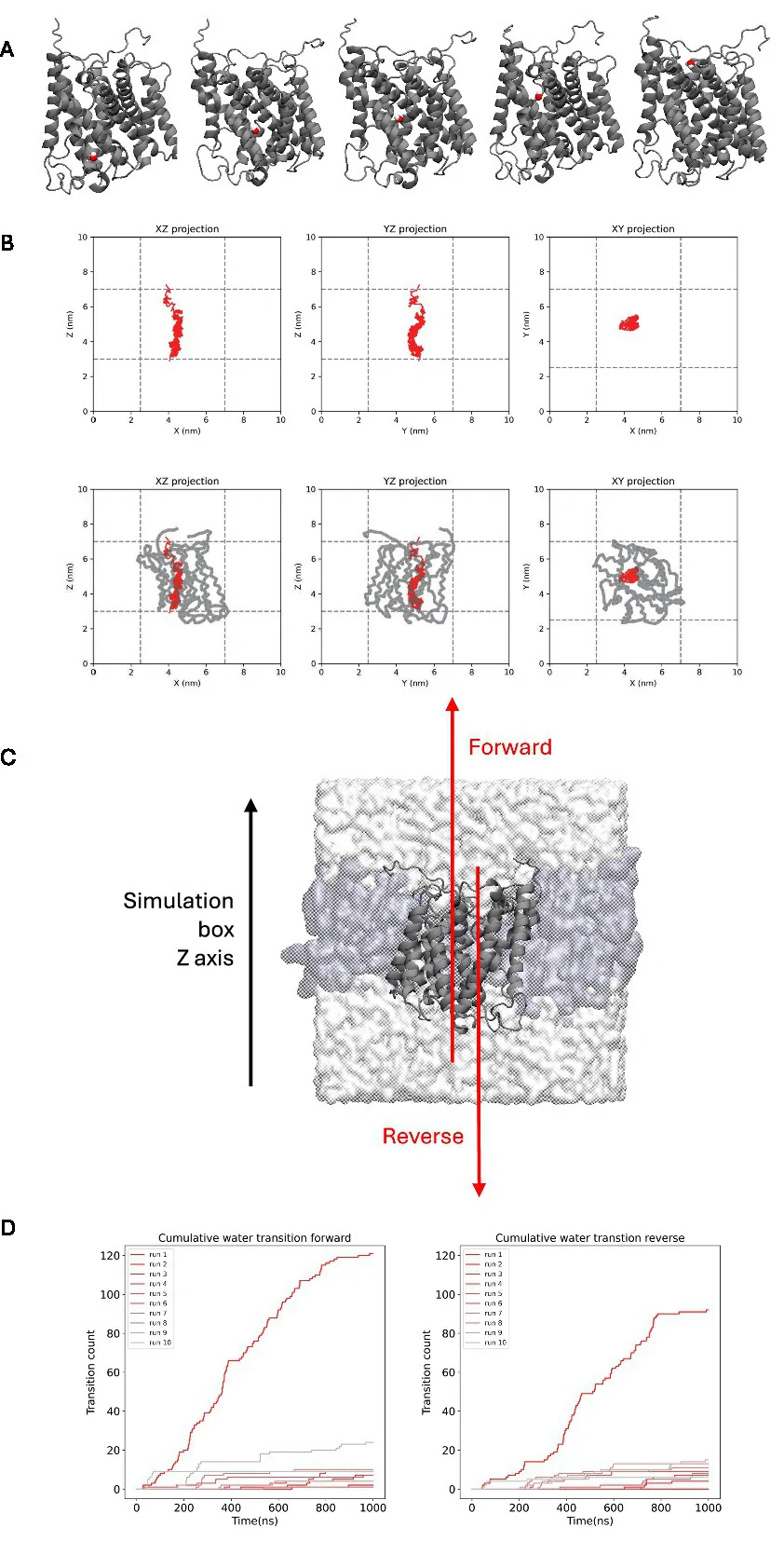
Water passage through GerAB during MD simulations. **(A)** One typical water passage event shown as a snapshot during MD simulations. GerAB protein is colored in grey and illustrated in new cartoon. The water molecule passing GerAB was illustrated in space filling, with the oxygen atom in red and hydrogen atoms in white. **(B)** Projection of the same water molecule passing through GerAB trajectory on XZ, YZ, and XY planes of the simulation box. Three projections of the water trajectories are shown to demonstrate that the water passages selected by our analysis indeed occur through GerAB, rather than penetrating the membrane. The top panel shows the water trajectory. The lower panel shows the water trajectory with protein backbone illustrated in grey thread. Dotted lines indicated the X, Y, and Z bound protein to calculate water passage. **(C)** Simulation box of the protein/membrane system in this study. Water and ions are depicted as a transparent surface, and the lipid bilayer is depicted as blue surface. The direction of the Z axis is shown as a black arrow, and the forward and reverse water passage directions are shown as red arrows. **(D)** Cumulative water passage profile of ten individual simulation runs in both directions.

We then carried out analyses with data from 200 ns onward, after the equilibrium indicated by RMSD. One typical water crossing event is captured in Figure 1A as snapshots of MD simulations, and the trajectory for this water molecule is projected on the simulation box in Figure 1B. The overall water permeation profiles from all ten production runs are summarized in Figure 1C. Across the ten simulations, two distinct patterns of water permeation emerged. In nine of the runs, water passage through the channel remained limited (between 1 to 24 molecules/μs), whereas one trajectory exhibited higher amount of water passage at 121 molecules/μs. This observation is consistent with a previous study on GerAB using the same simulation setup (Blinker et al., 2021), in which a pore radius of approximately 0.1–0.3 nm was reported across five parallel 100 ns MD simulations. To further strengthen our understanding of water occupancy within GerAB and to demonstrate that the inner cavity is geometrically sufficient to accommodate water molecules, we performed an additional analysis. Specifically, we quantified the number of water molecules along the Z coordinate of GerAB across the simulation trajectories. Across all ten simulation runs, seven showed water occupancy at all Z-coordinate levels, while three showed no water occupancy when Z at 5.5–6.5 nm. This indicates that although the cavity undergoes dynamic changes, it remains sufficiently large to accommodate water molecules (Supplementary Figure S2). Moreover, during all simulation trajectories, the permeation events happen in both directions, meaning water could enter from both sides of the protein. With the Z axis of the simulation box aligning with the principal axis of the protein, water entering from low Z coordinate and exiting from high Z coordinate was defined as “forward,” while the other direction was defined as “reverse” (Figure 1C). Throughout the simulations, the number of forward and reverse permeation events are at the same order of magnitude at all times (Figure 1D). This observation is expected, as the simulations were performed under equilibrium conditions with no concentration gradient across the membrane. Consequently, there is no driving force for water entering the protein other than diffusion. In other words, during the simulations, water movement in the “forward” direction is equally likely as movement in the “reverse” direction. The distribution of the time individual water molecules took to pass through GerAB peaks at 5 ns and exhibits a long tail extending to 20 ns (Supplementary Figure S3). Under the current simulation setting, GerAB’s structure and flexibility shows that it is permeable for water, with a mechanism of passive diffusion. Passive diffusion is a plausible mechanism under equilibrated simulation even when the hydration on either side of the membrane is different (which is most likely the case in a spore during germination). The observations we make can be extrapolated to low hydration of the inner spore, under the caveat that GerAB does not change conformation. It is also important to note that the “forward” direction is likely more relevant to the physiological context during spore germination, as water intake goes from the outside to the inside of spore during germination. In this study, we used equilibrated simulation conditions to examine the interaction between GerAB and water, with water present on both sides of the lipid bilayer rather than only on the extracellular side. This setup serves as a starting point for understanding GerAB–water interactions and provides a foundation for future non-equilibrated simulations. This can be realized in simulation systems with water present only outside the membrane that provides an osmotic gradient, along with no periodic boundary conditions along the Z direction. At the same time, the water passage events observed here occur in the apo (non–ligand-bound) starting structure of GerAB. This implies that other physiologically relevant process in GerAB, such as water transport coupled to ligand-induced conformational changes, is beyond the scope of this study.

### 2.2 Identification of residues interacting with passed water

To investigate the mechanism of water passing through GerAB, we identified residues that came into contact with water molecules during passing events that occurred in the simulations. The contact frequency f of each residue was computed by counting the number of frames where the passing water oxygen was within 3.5 Å (length of a typical hydrogen bond) of any heavy atom of the residue and divided by the total number of frames. We identified 16 residues that exhibited f above the statistical significance threshold of 0.01 in both passage directions (Supplementary Table S1). In the forward water passage direction, all passage water took a total sum of 1,285,813 simulation frames to traverse GerAB. A contact frequency of 0.01 of one residue therefore corresponds to a sum of 12,858 frames, indicating that the residue was in contact with water for a total of approximately 25.7 ns in a simulation time of 1 μs. To explore whether the contacts between permeating water molecules and residues are persistent or transient, we analyzed, for each residue, the duration of individual interactions with water. For all residues, water interactions are predominantly transient, typically lasting around 2 ps which is the interval between two consecutive frames. This indicates that contacts between water and high-contact residues are short-lived but recurrent (Supplementary Figure S4). This interaction pattern is consistent with water permeation or flow rather than stable water binding (Ozu et al., 2013).

**Figure 2:**
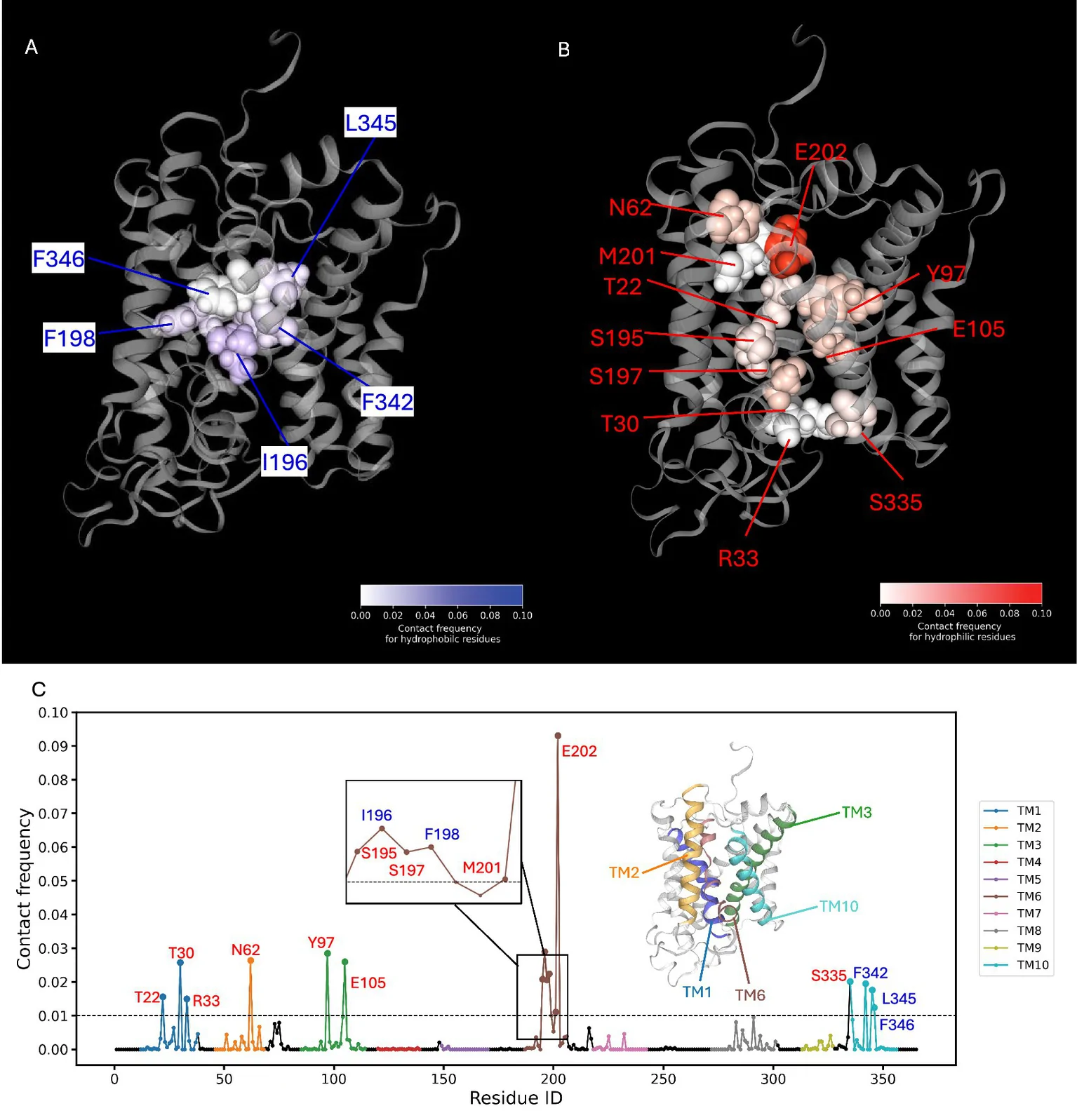
Identification of GerAB residues in contact with water. **(A,B)** High contact frequency hydrophobic/hydrophilic residues in GerAB. The protein backbone is illustrated as a grey ribbon, with high contact frequency residues highlighted in space filling according to their contact frequency f . Each individual residue is labelled in both subplots. **(C)** Contact frequency according to esidue ID and orward frequency $f_{F}$ are shown as a plot. The residues with a contact frequency above 0.01 in both directions are labelled. One snapshot of GerAB structure is inserted in this subplot with TM regions including high contact frequency residues represented in same colour in the plot. Hydrophilic residues are coloured in red and hydrophobic residues are coloured in blue in all subplots.

The water pathway, comprised of the residues in contact, is illustrated in Figures 2A,B with the residues coloured according to their contact frequency with water in different orientations of the simulation box. Based on this analysis, water was found to traverse specific regions of the protein, namely TM 1, 2, 3, 6, and 10, with less contact observed in TM 4, 5, 7, 8, and 9 (Figure 2C). Among these residues, 10 are hydrophilic (including glutamic acid, arginine, serine, threonine, asparagine and methionine) and 5 are hydrophobic (including isoleucine, leucine and phenylalanine). Among the 11 hydrophilic residues, 8 interacted with water via their side chains, while the remaining 2 made contact through backbone atoms (Supplementary Table S1). Water-contact residues identified here are not overlapping with the experimentally proven L-alanine binding pocket in GerAB that consists of G25, V101, L199, G200, T287, and Y291 (Artzi et al., 2021). Thus, these data indicate that water crossing in GerAB might *not* be stabilizing the binding pocket and facilitating ligand binding as reported in GkApcT (Afshinpour et al., 2023), otherwise the water path would go through the binding pocket. This inspired us to further verify the function of water-contact residues. Given water’s polar hydrogen-oxygen bonds and its capacity to form hydrogen bonds, we focused on verifying the functional relevance of the 8 hydrophilic residues that interact with water through their side chains. These were designated as “high-contact residues” and selected for *in silico* and *in vivo* validation.

### 2.3 High contact residue alteration alters water passage profile in simulation

**Figure 3:**
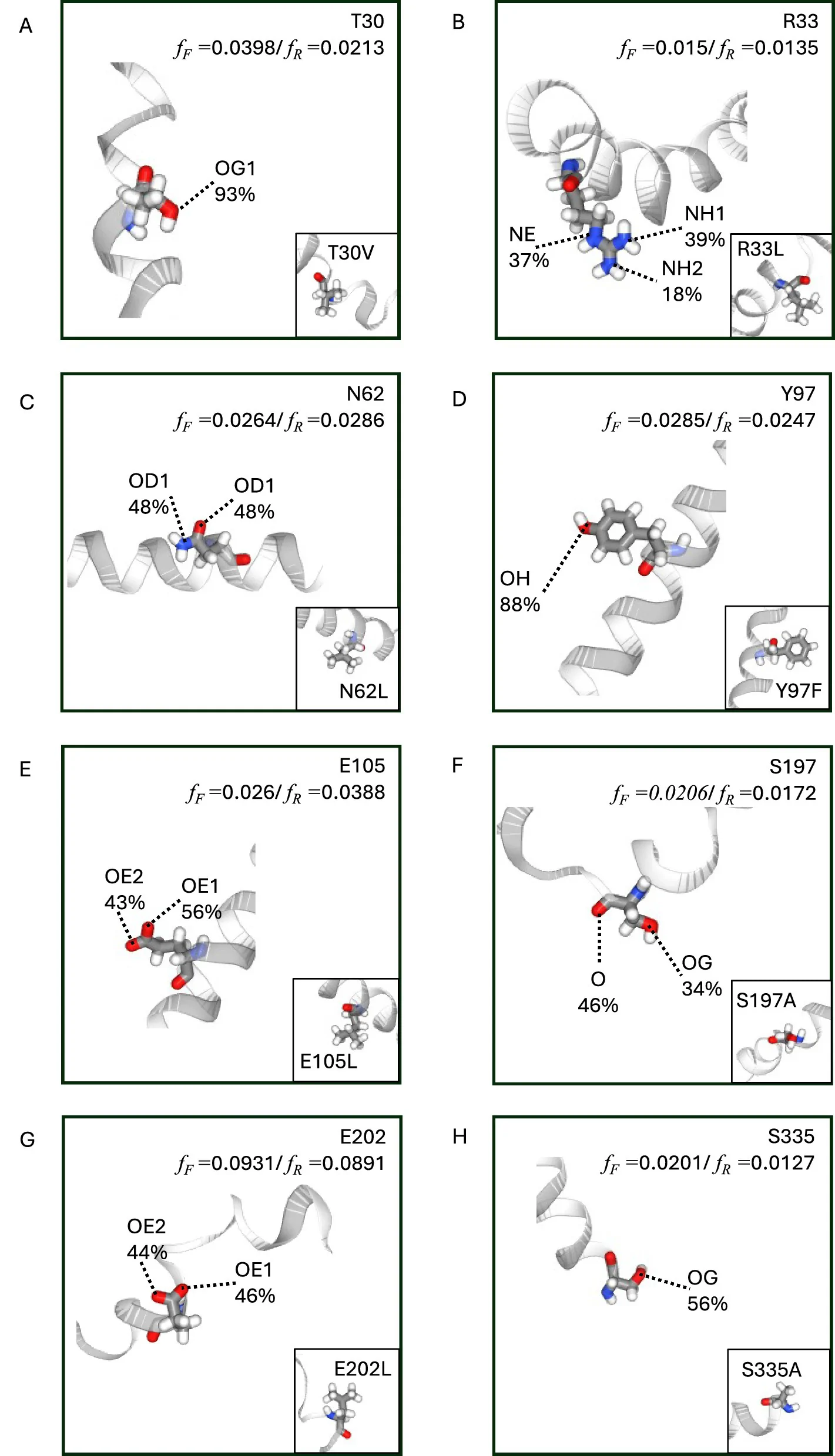
Substitution of high contact frequency hydrophilic residues. Each residue is depicted in one panel **(A–H)** in this figure with its $f_{F}$ and $f_{R}$ . The TM region of each residue is depicted to show the context of the protein. The protein backbone is depicted as a grey ribbon while selected residues are depicted in licorice representation, with oxygen in red, nitrogen in blue. The atom(s) with highest contribution of interactions between a residue and the passing water molecule(s) are labelled in each panel. The mutation introduced into GerAB was inserted at the bottom right corner in each panel.

Based on contact frequency analysis, we first incorporated single point mutations on high-contact residues *in silico* and tracked their water passage profile in simulations. Since drastic changes in side-chain size may negatively impact the stability of GerAB as reported previously (Cooper and Moir, 2011; Chen et al., 2025), target residues were mutated into residues with similar sized sidechains. This is done by computing, within each residue, which atom contacts water most frequently, represented as the percentage of total water-contact frames for that residue. The high-contact residues and their respective substitutions are illustrated in Figure 3.

We then set up MD simulations with GerAB single point mutants in the same set up as used for the wt GerAB-membrane system, see Methods for more details. For each variant of GerAB, five replicas were run. This was followed by analysing water permeation events for each simulation run. The water passage numbers for each mutant simulation run are shown in Table 1 (forward direction) and Supplementary Table S2 (reverse direction). Consistent with wild-type GerAB, the number of forward and reverse permeation events remained on the same order of magnitude in all systems, as the simulations were carried out under equilibrium conditions. Most of the mutants exhibited reduced water passage counts compared to the wild type simulations. Notably, some mutants, such as E202L, showed water permeation counts close to zero in both directions across all simulation replicates. This finding reinforces our analysis, as E202 was identified as the residue with the highest contact probability among all high-contact residues. On the other hand, the GerAB Y97F mutant displayed an increased level of water crossing ranging from 0 to 164 water molecule/μs. The elevated water passage observed in the Y97F mutant may be attributed to other factors, such as altered interactions among neighbouring residues, which require further characterization.

**Table 1:** Water crossing passage numbers of mutant GerAB simulations in the forward direction.

Mutant	Run 1	Run 2	Run 3	Run 4	Run 5
T30V	3	2	2	1	44
R33L	39	7	34	11	10
N62L	1	4	4	12	6
Y97F	1	0	61	164	0
E105L	1	2	0	0	23
S197A	42	6	2	21	1
E202L	1	0	0	1	0
S335A	0	0	1	16	3

Each mutant shows passage numbers per run.

### 2.4 High contact residue alteration impairs germination via structural disruption

**Figure 4:**
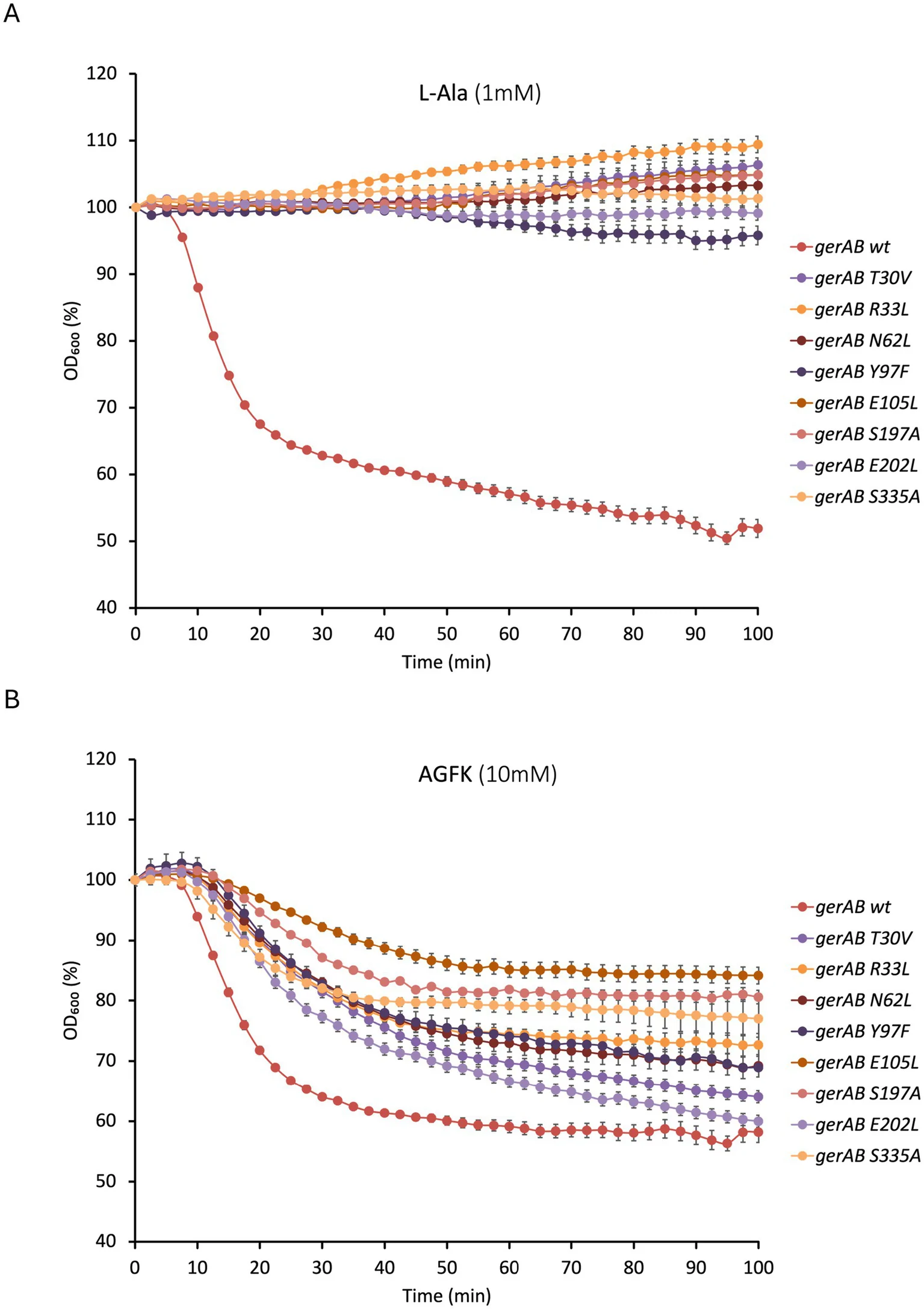
Germination assays of *B. subtilis* spores harboring wt and mutant GerAB genes with L-alanine **(A)** or the AGFK mixture **(B)**. As shown in **(A)**, all variants of mutant spores are unable to germinate with L-alanine. At the same time, all variants of mutant spores exhibited slow germination in response to the AGFK mixture, albeit to different extents **(B)**. Error bars indicate ± SD of three technical replicates.

**Figure 5:**
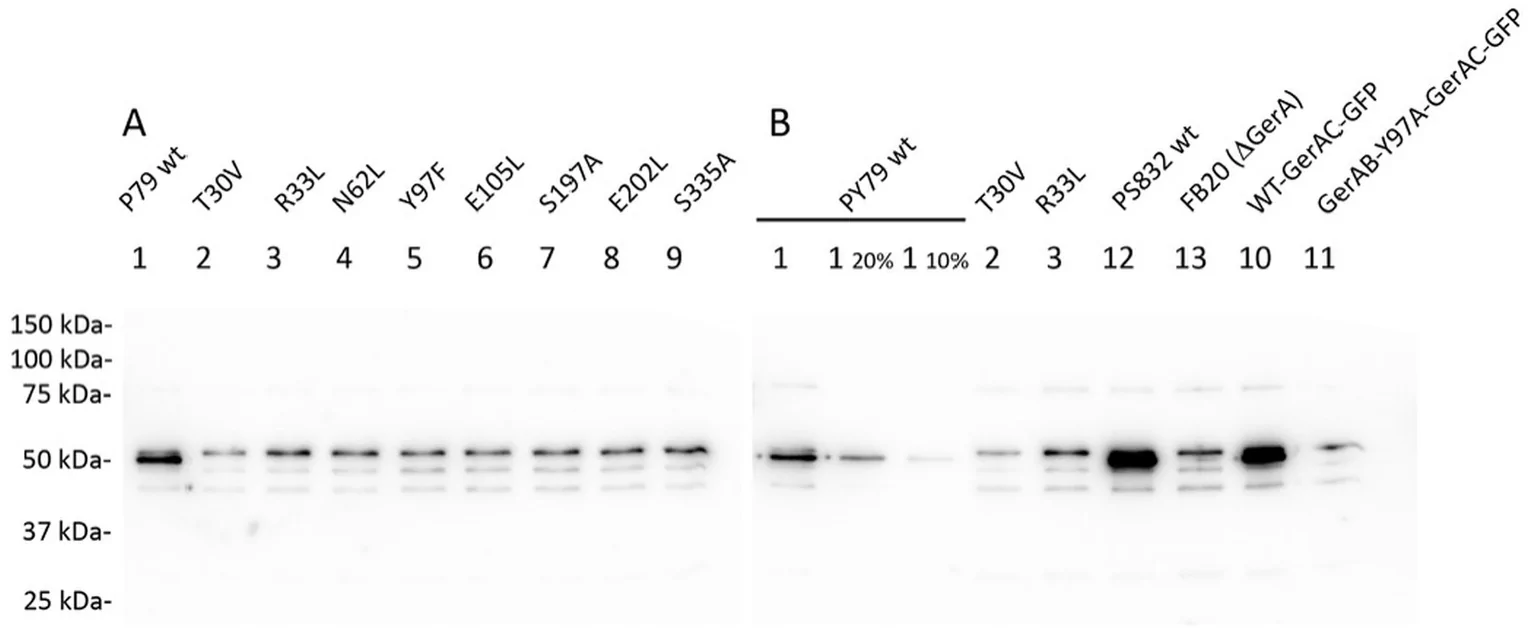
Western blots of spores harbouring different *gerAB* variants and stained with an anti-GerAA antibody. Specific bands ~50 kDa are labelled with red dots and varied in intensity between the lanes with wild-type strains PY79 and PS832. Band specificity was identified in panel **(B)** by comparison with a PS832 mutant, FB20, lacking the *gerA* operon and by titration of the analysed spore extract at 20 and 10% dilution of the starting material. Note, nonspecific bands included in the gerAB mutants are the ones slightly above the specific band **(A)**. The T30V and R33L gerAB mutants were included in the blot of panel **(B)** for comparison. Also, for comparison, spores containing the previously analysed PY79 mutant GerAB Y97A (Chen et al., 2025) with minor residual GerAA antiserum cross-reactive material were included as GerAB-Y97A-GerAC-GFP. GerAC-GFP alone in PY79 showed clear GerAA cross-reactive material. The reason PS832 wt and PY97 WT-GerAC-GFP show greater intensity of the GerAA band is because Coomassie staining of the gel from which the blot was made showed higher total protein levels.

To verify whether these high-contact residues contribute to water permeation *in vivo,* we constructed single point mutant spores with same mutations in simulations. The effect of each mutation was verified by tracking their germination kinetics. Figure 4A shows that all 8 mutants have reduced spore viability as they failed to germinate in the presence of L-alanine. To investigate whether mutant proteins were incorporated in the spore, we relied on the fact that the A and C subunits of the GerA GR depend on GerAB for stability (Cooper and Moir, 2011; Mongkolthanaruk et al., 2011). The detection of GerAA level was therefore employed as a method to detect folding of GerAB and the assembly of GerA. Immunoblot analysis showed in all the mutant spores that the levels of GerAA were similar to those of spores with Δ*gerA* (Figure 5). In addition, spores containing the previously analysed PY79 mutant GerAB Y97A (Chen et al., 2025) with minor residual GerAA antiserum cross-reactive material were included as GerAB-Y97A-GerAC-GFP. GerAC-GFP alone in PY79 showed clear GerAA cross-reactive material. The data show that spores harbouring any of the mutants made GerAB unstable or (close to fully)unable to form a complex with GerAA and GerAC. To assess if making mutations on other residues with functional importance on GerAB causes structural disruption, we carried on constructing mutants in the previously reported GerAB L-alanine binding pocket, G200A and G25A (Artzi et al., 2021). Contrary to prior findings, which showed that G25A forms a stable GerA complex, we observed neither of these two binding pocket mutants showed positive GerA assembly (Supplementary Figure S5). With this unexpected result, we examined the genome of the G25A mutant constructed in our lab. Since the gene construct used in this study to introduce single point mutations is based on a double-crossover strategy and contains the entire GerA operon (including its promoter), it is capable of being expressed independently of the insertion locus (Supplementary Figure S6). Our analysis showed two copies of the G25A*-gerA* operon instead of a single copy in the wt strain, indicating the gene copy number might play a determining role in GerA assembly. In summary, minor alterations to the GerAB protein on residues with high water-contact cause structural disruption of GerAB. However, changing binding pocket residue G25, which is not a high-water-contact residue, does not cause structural disruptions, according to the Western Blot in the original paper (Artzi et al., 2021). In addition to the putative L-alanine binding pockets, we observed that TM4, TM5, TM7, TM8, and TM9 exhibited relatively low contact frequencies with permeating water. These TMs are positioned on the outer layer of GerAB, compared to the water contacting residues locating on the core of GerAB (Figure 2). Notably, previously reported mutants, including F272I, F272A, D236A, D237A, and Y136F, located on TM8, TM9, and TM4, all of which showed positive GerAC signals in Western blot analyses indicating a successful GerAB fold and GerA assembly (Cooper and Moir, 2011). These observations suggest that residues located in regions with limited water interaction, particularly on outer transmembrane helices, may be more tolerant to mutagenesis. This means residues located on TM4, 5, 7, 8,9, are therefore likely to represent structurally robust sites where substitutions do not severely disrupt receptor integrity. To further confirm the structural role of the identified high-water contact residues, we checked if they are conserved in the germinant receptor subunits of other *Bacillus* spore-forming species. With CLUSTAL Omega (Madeira et al., 2024), we aligned the amino acid sequences of GerAB with those of GerBB, GerKB from *B. subtilis*, GerVB from *Bacillus megatarium* QM B1551 and GerRB from *B. cereus* ATCC14579. This sequence alignment indicated that Y97 and E202 are fully conserved, while the other residues are not conserved in the other germinant receptor subunits (Supplementary Figure S7). Therefore, Y97 and E202 might have a conserved structural role in GRs of spore formers beyond *B. subtilis*, while the other residues might have more specific structural/ functional role in GerAB. Combining all this evidence, we conclude the high water-contact residues can be considered hotspots for maintaining GerAB structural integrity. Since none of the high water-contact residue mutants form a GerA complex that is detectable at close to wild-type levels on Western blot using cross-reactivity with a well characterised GerAA antibody as a proxy, it remains experimentally unproven whether there is water intake through GerAB.

To further examine whether the GerAB mutations on high-contact residues impact the structure and function of other GRs in the germinosome, we performed germination assays of mutant spores against germinants that are sensed cooperatively by the GerB and GerK GRs, namely the AGFK mixture. All mutants displayed reduced germination kinetics in response to AGFK, with varying degrees of impairment compared to wild-type spores (Figure 4B). Since GerB and GerK colocalize in the germinosome along with GerA, this suggests the function of GerB and GerK is dependent on the structural stability and full functionality of GerA, as mediated by GerAB. This was also observed in a previous study (Chen et al., 2025), and aligns with the current understanding of germinosome function(Griffiths et al., 2011; Wen et al., 2019). It is also consistent with the proposed model of subunit exchange between germinant receptors (as discussed by Prof. David Rudner at the 11th European Spore Conference, 2025), in which different GerAB mutants may assemble with GerB or GerK to varying degrees, resulting in distinct AGFK germination kinetics. However, confirming this hypothesis will require further investigation of the binding affinities between GR subunits, both within the same receptor complex and between different receptor complexes.

## 3 Discussion

With MD simulations, we identified water passage events through GerAB and further identified 8 hydrophilic residues in GerAB that exhibit high contact frequency with water during all permeation events. Altering these residues to similar sized hydrophobic residues altered water passage *in silico*, with most of the mutants showing decreased water passage in MD simulations. The *in vivo* role of these residues was verified with mutagenesis followed by germination assay with different germinants. All 8 mutants showed severe germination defects with L-alanine and decreased germination with the AGFK mixture. With Western Blot showing no successful assembly of GerA receptors in all mutant spores, the L-alanine germination defect was caused by the absence of the GerA GR. This indicates the identified high water contact residues are also hotspots for GerAB structural integrity.

The role of water in GerAB observed in this study differs from that reported for other APC transporters, where water facilitates ligand binding (Afshinpour et al., 2023). A previous study identified the L-alanine binding pocket of GerAB involving G25, V101, L199 G200, T287 and Y291 (Artzi et al., 2021), yet in our current simulation setup, the water permeation path does not pass through this predicted pocket. This divergency suggests water permeation in GerAB may not contribute directly to ligand binding. Given the current experiment setup of this study, we are unable to determine whether GerAB facilitates water passage to promote germination. Instead, with the constructed mutants, we found that the disruption in GerA assembly also disrupted the structure of the GerB and GerK GR which directly reflect germinosome assembly, as observed in a previous study (Chen et al., 2025). To further prove the structural co-dependency between GRs, future studies could focus more on the affinity for the cognate A and C subunit partners in GerA, GerB and GerK.

Given that germinant receptors are directly linked to the initiation of bacterial spore germination, these findings extend beyond receptor/germinosome structural characterization. A broader significance of this study lies in its potential application to spore control strategies. Since bacterial spores are highly resistant to environmental stress and are responsible for food spoilage and spore-mediated diseases, understanding the molecular determinants of germination opens possibilities for “germinate to eradicate” approaches for spore control (Christie and Setlow, 2020). By artificially triggering germination under controlled conditions, spores can be altered to be more susceptible to conventional sterilization or antimicrobial treatments. Thus, the mechanistic insights provided here may inform future strategies aimed at combating the persistence and impact of spore-forming bacteria in food safety and public health concerns.

## 4 Methodology

### 4.1 MD simulations of GerAB-membrane systems

The GerAB-membrane system was prepared according to Blinker et al. (2021). In short, GerAB structure was predicted by the RaptorX structural prediction tool (Källberg et al., 2012), and the structure was used subsequently to construct a protein-bilayer membrane system and introducing mutations using the CHARMM-GUI (Lee et al., 2016), with a bilayer membrane composition mimicking the spore IM (with the lipid ratio of POPE: POPG: TMCL = 1:6:3). The system was solvated by the TIP3P water model, as CHARMM36 forcefield is parametrized with TIP3P. 20 mM KCl was used to neutralize the system. MD simulation of the GerAB-membrane system was carried out using GROMACS engine, version 2023.3. We used the (Aguayo et al., 2012; Lee et al., 2016), and the long-distance electrostatic interactions were computed by using aparticle mesh Ewald (PME) algorithm (Cheatham et al., 1995) with a grid spacing of 0.12 nm. The temperature was kept at 298 K with the velocity rescaling thermostat (Bussi et al., 2007) and the pressure was kept constant around 1.0 bar using the Parrinello and Rahman (1981). To remove any steric clashes and hindrances, the systems were first energy minimized using the steepest descent algorithm (Hoover, 1985), followed by equilibration carried out according to Lee et al. (2016). The production runs were 1,000 ns each, using a 2 fs timestep with frames saved every 2 ps. To avoid excessively large output files, trajectories were generated in segments of 100 ns each. 10 parallel 1 μs simulations were performed under identical conditions for the wt system, and 5 parallel simulations were performed for mutant systems. Each individual simulation was initiated with different random velocities. All the production runs were carried out under periodic boundary conditions (PBC). Details of the MD simulation setup are provided in Table 2.

**Table 2:** Detail of MD simulations systems.

GerAB variant in simulation system	No. of water molecules	No. of K^+^ ions	No. of Cl^−^ ions	Temp. (K)	No. of repeats
wt	19,336	236	6	298	10
T30V	19,321	236	6	298	5
R33L	19,291	237	6	298	5
N62L	19,319	236	6	298	5
Y97F	19,321	236	6	298	5
E105L	19,285	235	6	298	5
S197A	19,325	236	6	298	5
E202L	19,322	235	6	298	5
S335A	19,316	236	6	298	5

### 4.2 Water permeation analysis

The stability of the simulation system was monitored by measuring with root-mean-square deviations and root-mean-square fluctuations (RMSF) of the protein carbon alpha (CA) atoms. During simulations, one water permeation event was defined as a water molecule increasing its Z coordinate in each consecutive frame after it reaches the entrance of the protein until it exited the protein, while remaining within the cylinder of protein. If a water molecule goes through the entrance and exits in two consecutive frames (2 ps), it is considered to go through a periodic boundary jump and it is not considered as a valid permeation event. The entrance and exit of the protein along the z axis were defined as *Z* = 2.5 nm and *Z* = 7.5 nm, respectively. The cylinder of the protein was defined between 3.5 nm and 6.5 nm for both x and y coordinates. Since the production runs are carried out under equilibrated conditions, water should permeate through both directions. Therefore, all the water molecules were examined in both directions and counted to give the total water passage, and all 10 runs for each system were analysed per 100 ns.

### 4.3 Water occupancy analysis

To analyse water occupation in GerAB, we divided the system into bins along the *Z*-coordinate and counted the number of water molecules within each slab. A slab size of 0.5 nm was used to define water-accessible regions, reflecting not only the ~0.3 nm size of a water molecule but also the additional space required for dynamic motion and hydrogen-bonding interactions during permeation. Water occupancy was measured for every simulation frame as the number of water molecules in each slab. For each simulation, the probability distribution of water occupancy in each slab was calculated to reflect the general accessibility of water molecules during the simulation.

### 4.4 Residue contact analysis

To determine the pathway traversed by water molecules during simulations, we computed the frequency of each residue i that came into proximity with permeating water, defined as f . To be specific, we first entailed recording the trajectory of all water molecules engaged in permeation events. For each simulation frame encompassing a water permeation event, we recorded the amino acid side chain heavy atoms within a 3.5 Å radius of the water oxygen atoms. To avoid double counting, only the closest heavy atom was counted as 1 contact. Subsequently, this count was normalized by dividing it with the total number of frames (1,285,813 in the forward direction and 1,109,025 in the reverse direction) encompassing all water permeation events. For each residue in contact with water, the contact frequency of each heavy atom was calculated and normalized by the total contact of that residue. Residue–water contact time was calculated for each high-contact residue. A contact was defined when the oxygen atom of a permeating water molecule was within 3.5 Å of any heavy atom in the side chain of the residue. The duration of such a contact event was then recorded. Residue–water contacts were quantified for both directions of permeation. Following the outlined procedure, all contacting residues were visually inspected using VMD.

### 4.5 Mutagenesis

To focus on biologically relevant interactions of residues in contact while minimizing noise from transient contacts, a frequency threshold of 0.01 statistical significance was applied in residue contact analysis for both permeation direction. Resides selected for mutagenesis are: (i) hydrophilic, (ii) reached 0.01 or more contact both directions, and (iii) the highest contact atom is a side chain atom. For the selected residue, the atom with the highest contact was examined and the residue was altered to the structurally closest residue without the highest contact heavy atom.

All strains were derived from *Bacillus subtilis* PY79. The *gerA* operon was cloned by PCR, inserted in plasmid vector pUC19 with the Gibson Assembly Master Mix kit (New England BioLabs, NEB # E2611S). Plasmid- based mutagenesis was carried out with the QuikChange Lightning Site-Directed Mutagenesis Kit (Agilent Technologies, Cat # 210518–5). Mutant *gerAB* sequences were integrated into the original *gerAB* locus by a double crossover along with an erythromycin resistance cassette. The correct sequence of the mutant *gerAB* locus was confirmed by DNA sequencing. Mutant strains established in this study are listed in Table 3.

**Table 3:** Strains used in this study.

Strain	Origin strain	Source
gerAB *Wild Type (wt)*	*Bacillus subtilis* PY79	Lab stock
gerAB wt-GerAC-GFP	*Bacillus subtilis* PY79	Constructed in this study
gerAB-Y97A-GerAC-GFP	*Bacillus subtilis* PY79	Constructed in this study
gerAB T30V*::erm*	*Bacillus subtilis* PY79	Constructed in this study
gerAB R33L*::erm*	*Bacillus subtilis* PY79	Constructed in this study
gerAB N62L*::erm*	*Bacillus subtilis* PY79	Constructed in this study
gerAB Y97F*::erm*	*Bacillus subtilis* PY79	Constructed in this study
gerAB E105L*::erm*	*Bacillus subtilis* PY79	Constructed in this study
gerAB S197A*::erm*	*Bacillus subtilis* PY79	Constructed in this study
gerAB E202L*::erm*	*Bacillus subtilis* PY79	Constructed in this study
gerAB S335A*::erm*	*Bacillus subtilis* PY79	Constructed in this study
gerAB G25A*::erm*	*Bacillus subtilis* PY79	*gerAB* S335A*::erm*
gerAB G200A*::erm*	*Bacillus subtilis* PY79	Constructed in this study
gerAB *Wild Type (wt)*	*Bacillus subtilis* PS832 (Paidhungat and Setlow, 2000)	Prof. Peter Setlow lab, UConn Health
ΔgerA	*Bacillus subtilis* PS832 (Paidhungat and Setlow, 2000)	Prof. Peter Setlow lab, UConn Health

### 4.6 Sporulation and spore purification

Bacterial strains were streaked on LB agar with the appropriate antibiotics and incubated overnight. A single colony was inoculated into liquid LB with antibiotics and grown to an OD₆₀₀ of 1.0–2.0, after which 200 μL of culture was spread on 2 × SG agar plates (Difco Nutrient Broth 16 g/L, KCl 26 mM, MgSO₄ 2 mM, MnCl₂ 0.1 mM, FeSO₄ 1.08 μM, Ca(NO₃)₂ 1 mM, Glucose 5.5 mM, Agar 15 g/L) without antibiotics. Plates were incubated upside down at 37 °C in plastic bags for 2–5 days for full sporulation as monitored by phase-contrast microscopy. Plates were then air-dried for ~2 days to promote cell lysis, and spores were scraped into cold Milli-Q water. Suspensions were sonicated for 1 min at full power, cooled on ice, and centrifuged at ~8,000 rpm for 20 min to remove debris. Spore purity was confirmed by phase-contrast microscopy.

### 4.7 Germination assay with optical density drop

Purified phase-bright spores were normalized to an OD₆₀₀ of 1.2 in 25 mM HEPES buffer (pH 7.4), heat-activated at 70 °C for 30 min, and cooled on ice for 20 min. Aliquots (100 μL) of heat-activated spores were dispensed into a 96-well plate, and germinants were added to final concentrations of 1 mM L-alanine or 10 mM AGFK. OD₆₀₀ was recorded every 2.5 min for 100 min at 37 °C with continuous agitation between readings. OD reduction was calculated relative to the initial value. Each assay was performed in triplicate, and mean OD drops are reported.

### 4.8 SDS-PAGE and immunoblotting

For western blotting, spores were decoated and lysed as follows. 50 OD units of spores were pelleted by centrifugation, resuspended in 1 mL TUDSE buffer (8 M urea, 50 mM Tris–HCl pH 8.0, 1% SDS, 50 mM DTT, 10 mM EDTA), and incubated at 37 °C for 45 min. After centrifugation (3 min, max rpm, room temperature), the pellet was resuspended in 1 mL TUDS buffer (8 M urea, 50 mM Tris–HCl pH 8.0, 1% SDS) and incubated again for 45 min at 37 °C. The spores were washed six times by centrifugation and resuspended in 1 mL TEN buffer (10 mM Tris–HCl pH 8.0, 10 mM EDTA, 150 mM NaCl). Decoated spores were stored in water if not processed immediately. For lysis, 50 OD units of decoated spores were treated with 1 mg lysozyme in 0.5 mL TEP buffer (50 mM Tris–HCl pH 7.4, 5 mM EDTA) containing 1 mM PMSF, 1 μg RNase, 1 μg DNase I, and 20 μg MgCl₂ at 37 °C for 6–8 min, followed by 20 min on ice. Glass disruptor beads (0.10–0.18 mm, 100 mg) were added, and spores were sonicated in three 10 s bursts (medium power, microprobe) with 30 s cooling on ice between bursts. After settling for 15 s, 100 μL of supernatant was mixed with 100 μL 2 × Laemmli buffer (5% 2-mercaptoethanol, 1 mM MgCl₂) and boiled at 90–95 °C for 3 min to obtain the total lysate. Twenty micrograms of total protein per lane were separated on a 10% SDS-PAGE gel (BIO-RAD #4561034) at 60 V for 45 min, then 110 V for 45 min, and transferred to a 0.22 μm PVDF membrane. The membrane was blocked with 2.5% low-fat milk in TBST (20 mM Tris, 150 mM NaCl, 0.1% Tween-20) for 30 min, incubated overnight with anti-GerAA antibody (1:3000) (20), washed three times with TBST, and probed with goat anti-rabbit HRP antibody (1:2500; BIO-RAD #1706515) for 1 h before visualization.

## Data Availability

The datasets presented in this study can be found in online repositories. The names of the repository/repositories and accession number(s) can be found in the article/Supplementary material.
