# Correction: GerAB residues predicted to interact with water based on MD simulations mediate germinosome stability in *Bacillus subtilis* spores

**DOI:** 10.3389/fmicb.2026.1963750

**Published:** 2026-09-03

**Authors:** Longjiao Chen, Silke van Buuren, George Korza, Jocelyne Vreede, Peter Setlow, Stanley Brul

**Affiliations:** 1 Swammerdam Institute for Life Sciences, University of Amsterdam, Amsterdam, Netherlands; 2 Van't Hoff Institute for Molecular Sciences, University of Amsterdam, Amsterdam, Netherlands; 3 Department of Molecular Biology and Biophysics, UConn Health, Farmington, CT, United States

**Keywords:** APC transporters, bacterial spores, germinant receptor, MD simulations, spore germination

The **Acknowledgments** section was omitted from the publishing article. The statement is as follows:

“The authors wish to thank MSc. Xiaoyi Li (Swammerdam Institute for Life Sciences, University of Amsterdam) for constructing the GerAC-GFP strain employed in this work. This strain was instrumental to us in enabling analyses of germinant receptor presence in Y97A mutant spores and germinosome integrity in the wt-strain PY79 background. Both are presented in this manuscript. The China Scholar Scholarship Council is acknowledged for its support with scholarship No. 202308310017 for MSc Xiaoyi Li and No. 202109120009 for MSc Longjiao Chen.”

In the published article, affiliations 2 and 3 were erroneously swapped. In the corrected article, affiliation 3 is now “Department of Molecular Biology and Biophysics, UConn Health, Farmington, CT, United States” (previously listed as affiliation 2), and affiliation 2 is now “Van't Hoff Institute for Molecular Sciences, University of Amsterdam, Amsterdam, Netherlands” (previously listed as affiliation 3).

The original version of this article has been updated.

