# Supplementary material for: GerAB residues predicted to interact with water based on MD simulations mediate germinosome stability in Bacillus subtilis spores
Source: Front Microbiol. 2026 Apr 29;17:1800262. doi: 10.3389/fmicb.2026.1800262 (PMC13167987; doi:10.3389/fmicb.2026.1800262)
Supplement: Supplementary file 1 [file Data_sheet_1.pdf]

Supplementary Information

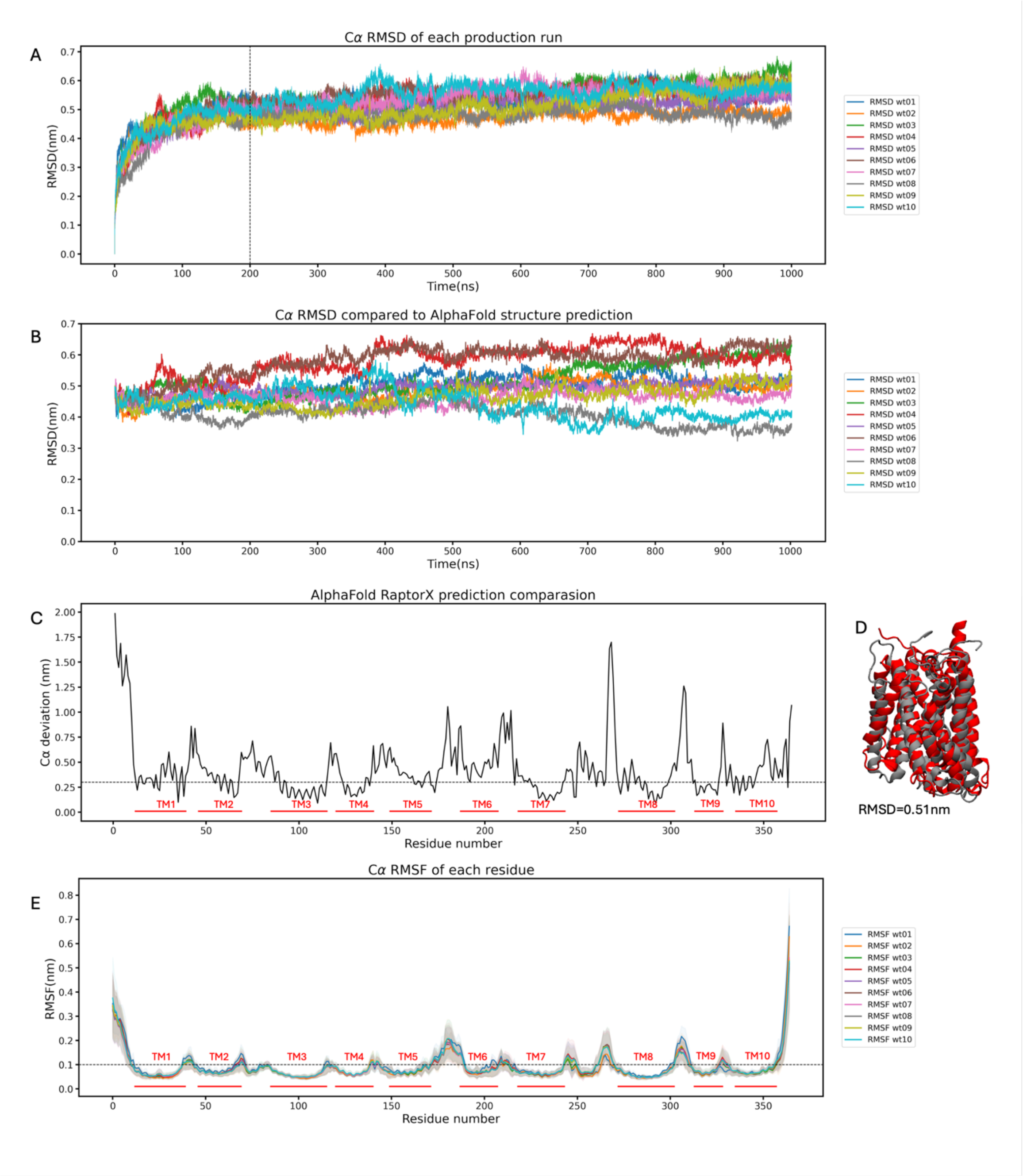

Figure S1. Protein structural stability during ten parallel MD simulations production runs. Protein CA RMSD (A) increased from 0 to 200ns simulations and stabilized afterwards at around 0.6nm in all 10 simulation runs. Protein CA RMSD in reference to AlphaFold structure prediction (B) remained stable around 0.5nm in all 10 simulation runs. (C) Comparison of AlphaFold and RaptorX structural predictions represented by per-residue CA deviations. Transmembrane (TM) regions are indicated by red bars. The two models exhibit similar overall folds, with low deviations in TM regions and higher deviations at the termini and inter-TM loops. (D) Structural overlay of the AlphaFold prediction (red) and the RaptorX prediction (grey), with a overall RMSD at 0.51nm. Protein CA RMSF (E) showed the RMSF of each residue calculated per 100ns and averaged over each production run. Error margin showed standard deviation of RMSF of each residue. All TM regions were marked as red bars in the figure.

(A)

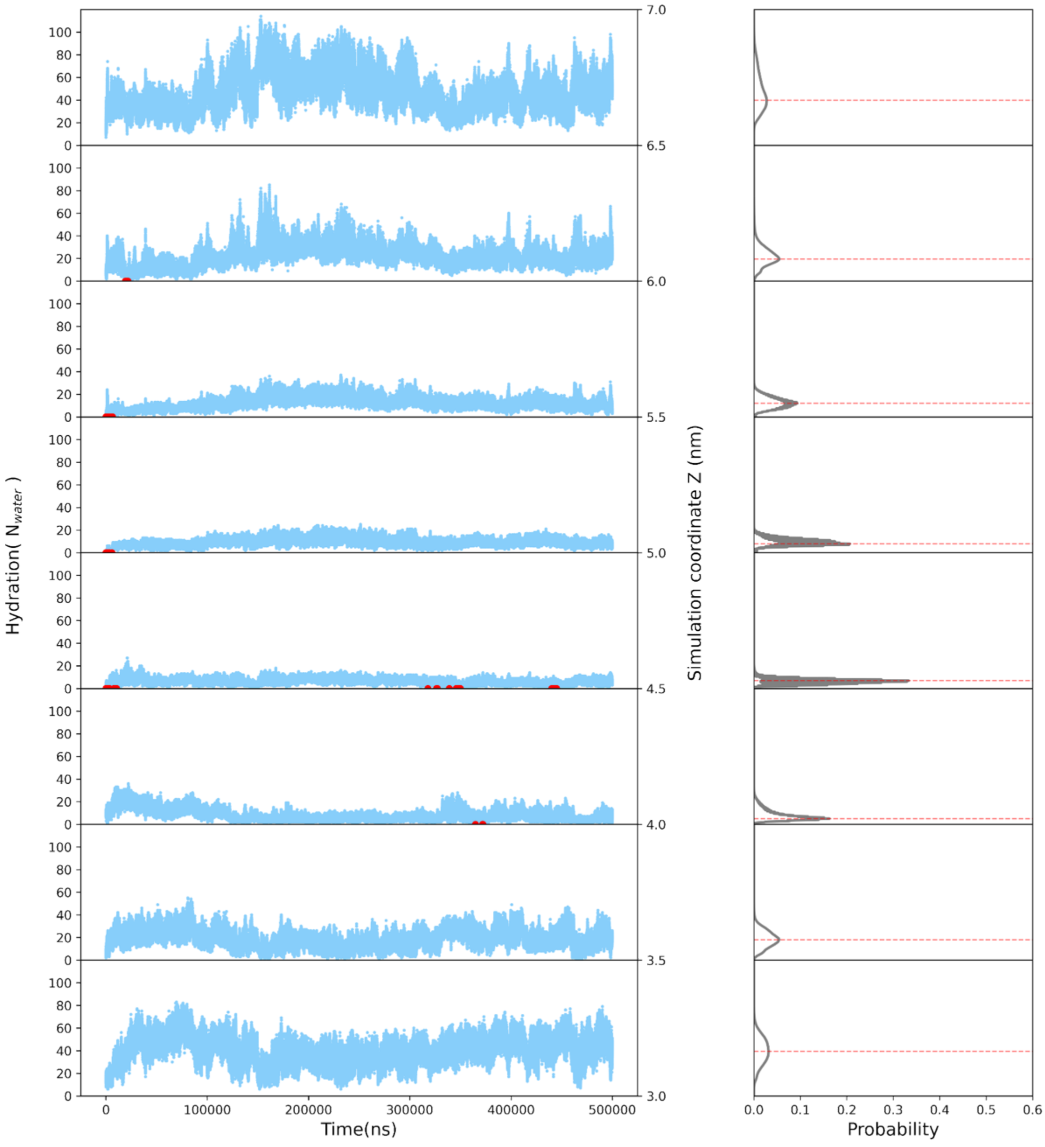

This Figure continues in next page

(B)

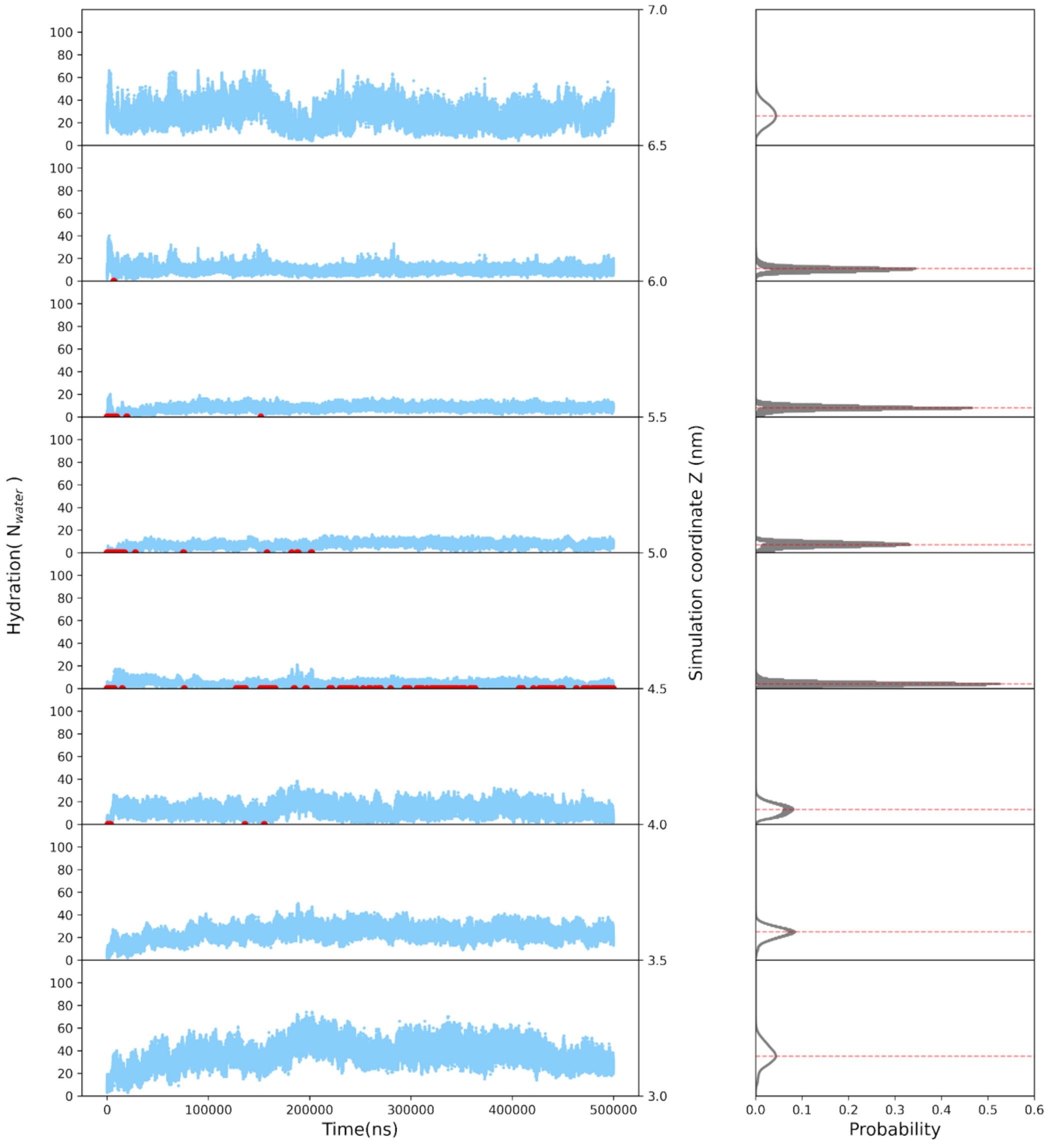

This Figure continues in next page

(C)

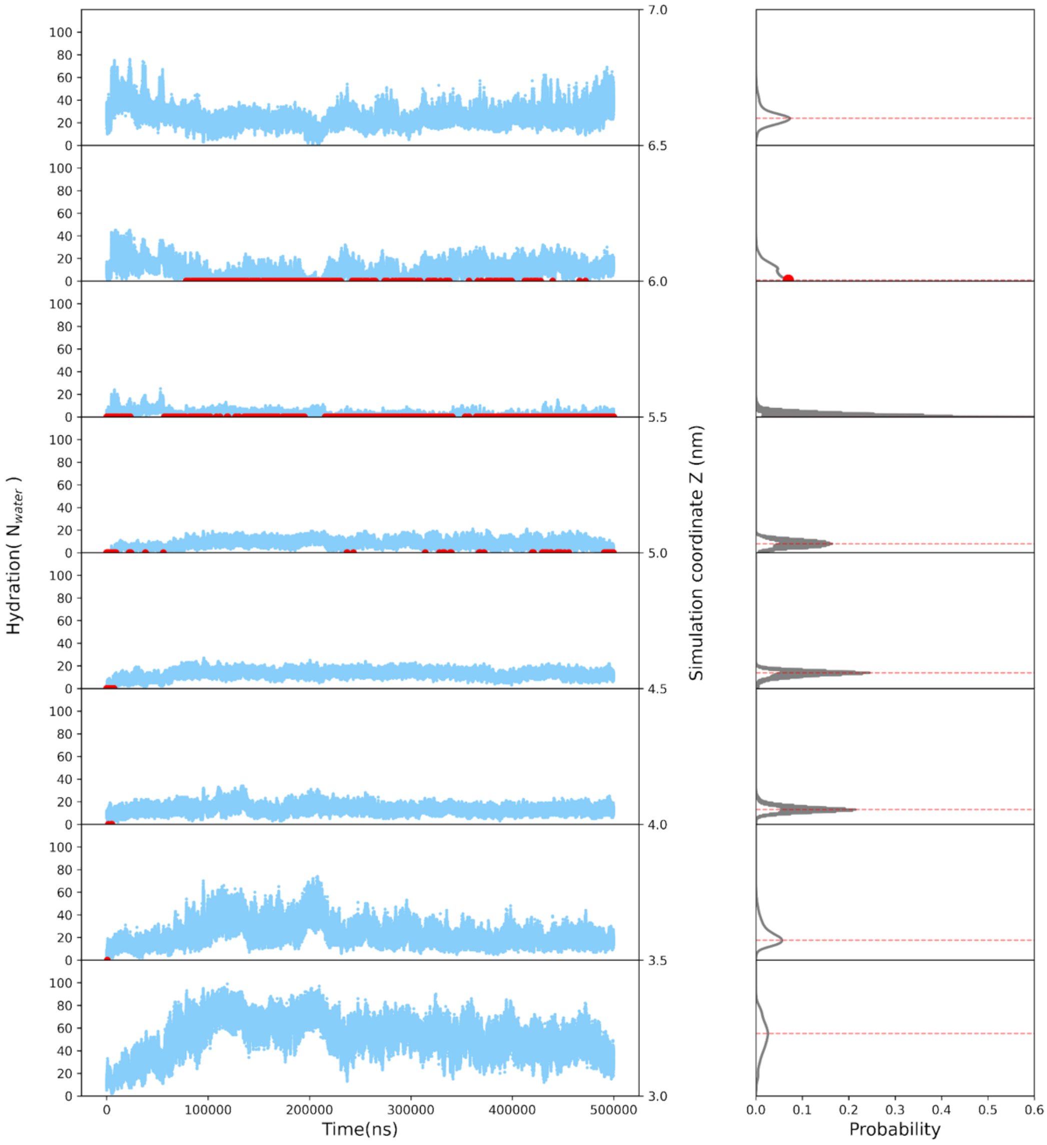

This Figure continues in next page

(D)

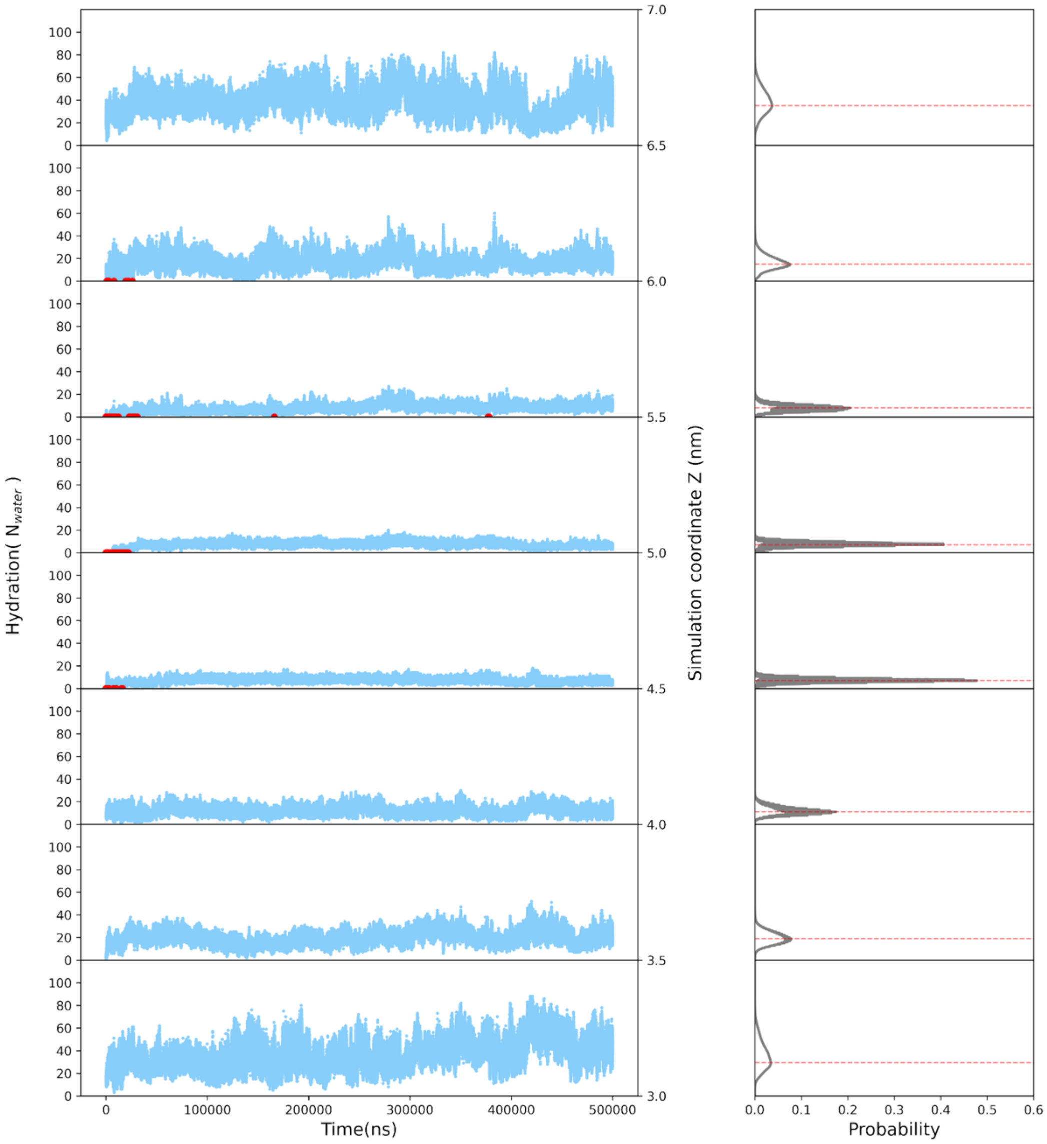

This Figure continues in next page

(E)

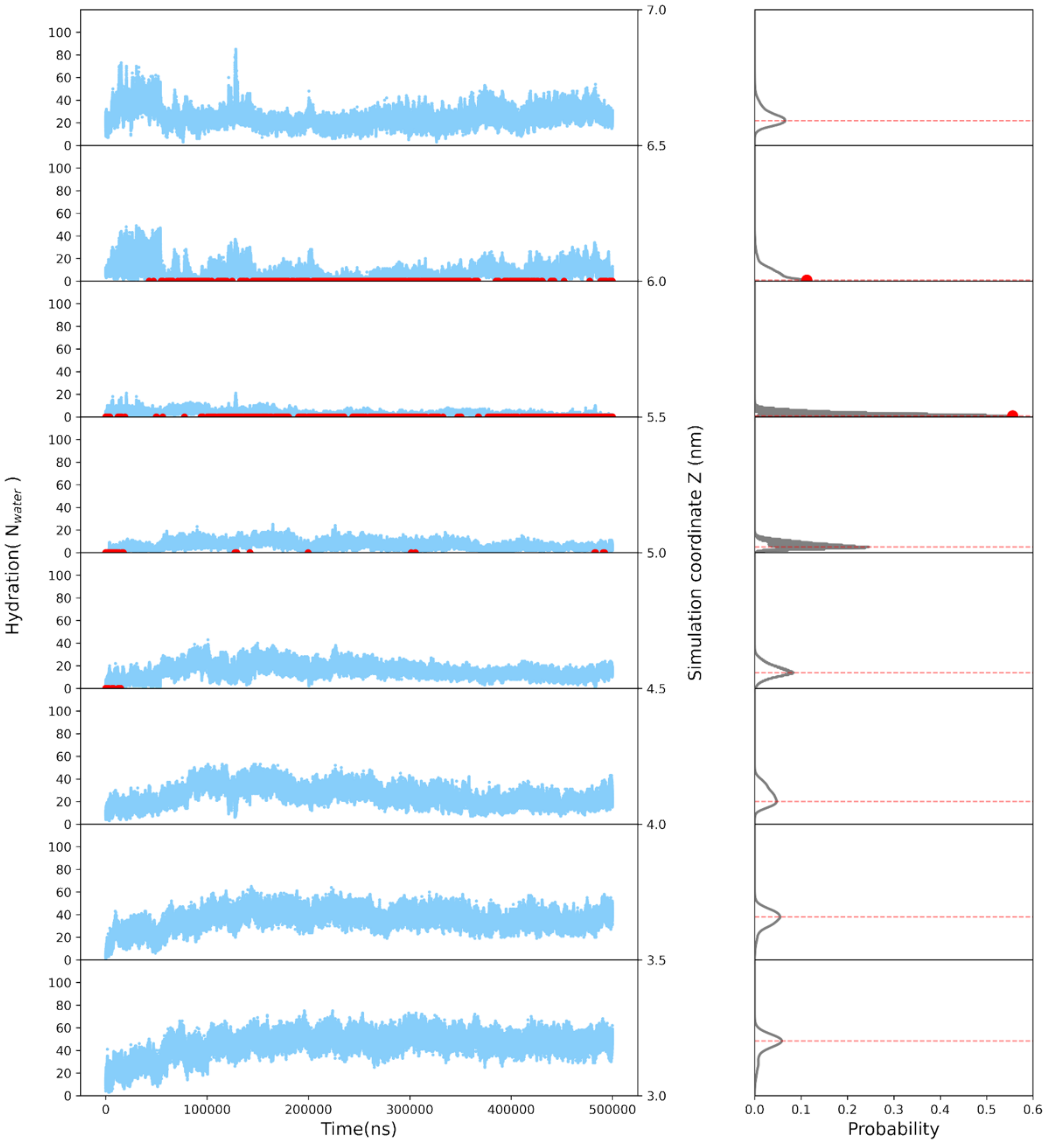

This Figure continues in next page

(F)

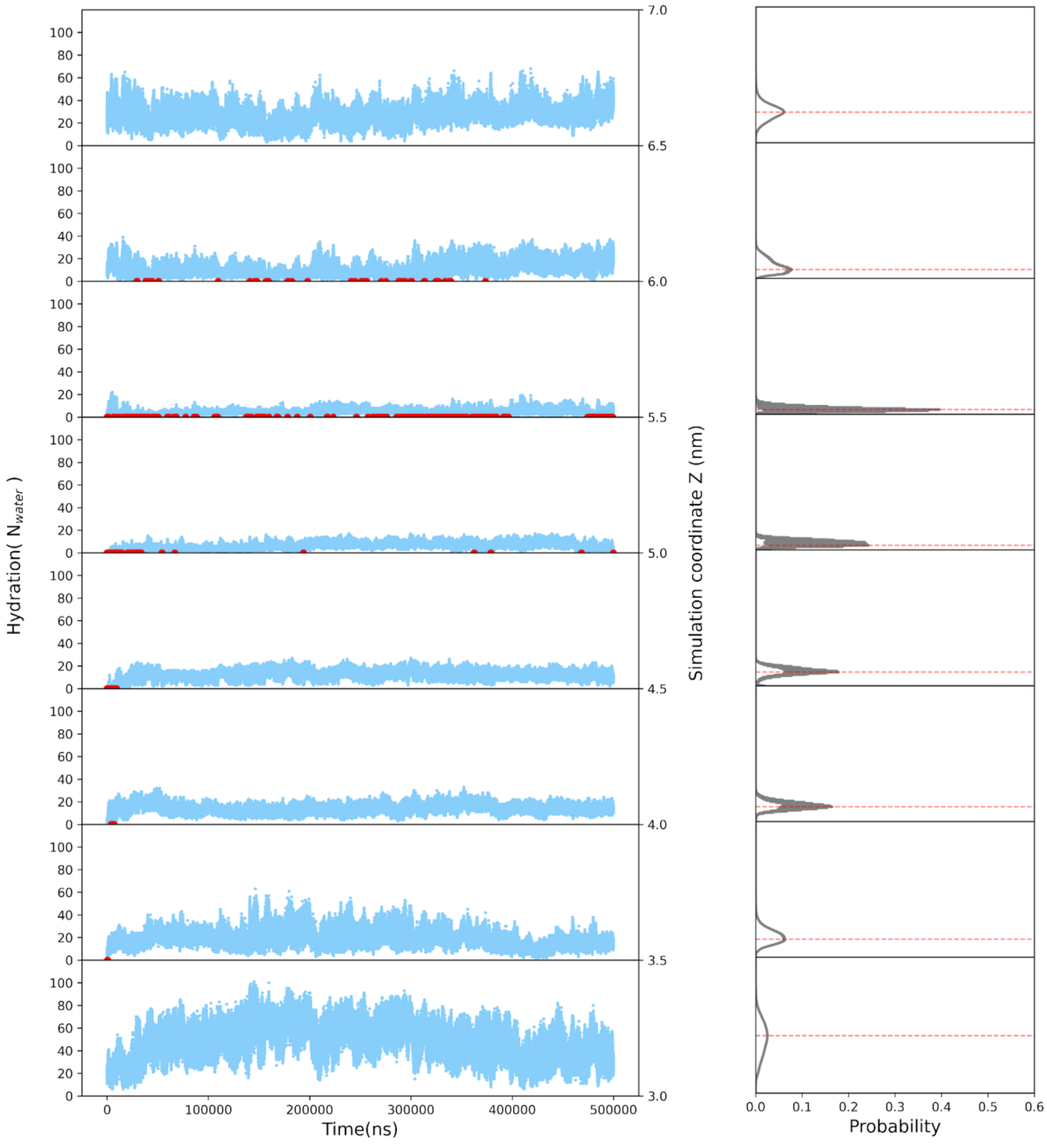

This Figure continues in next page

(G)

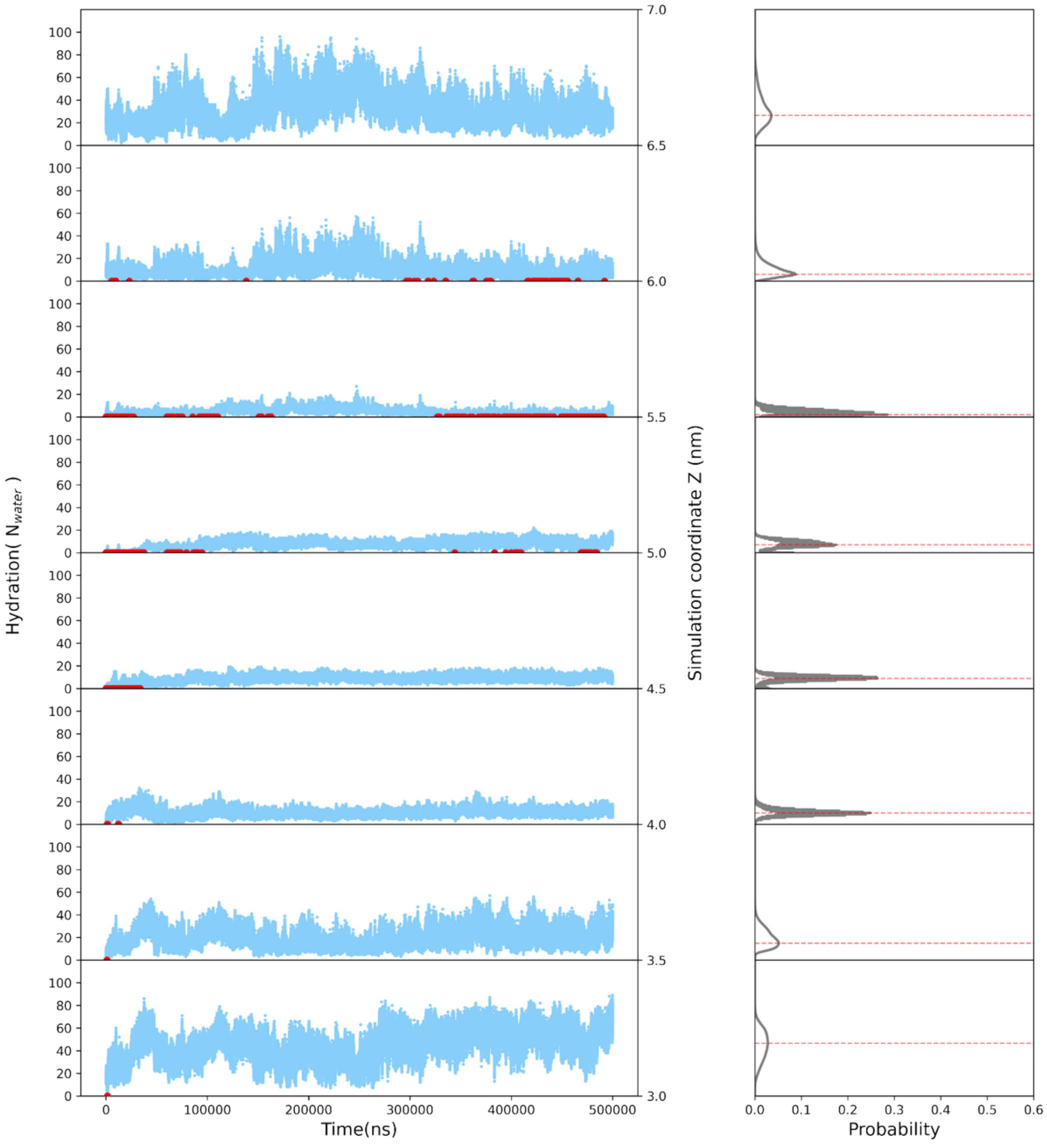

This Figure continues in next page

(H)

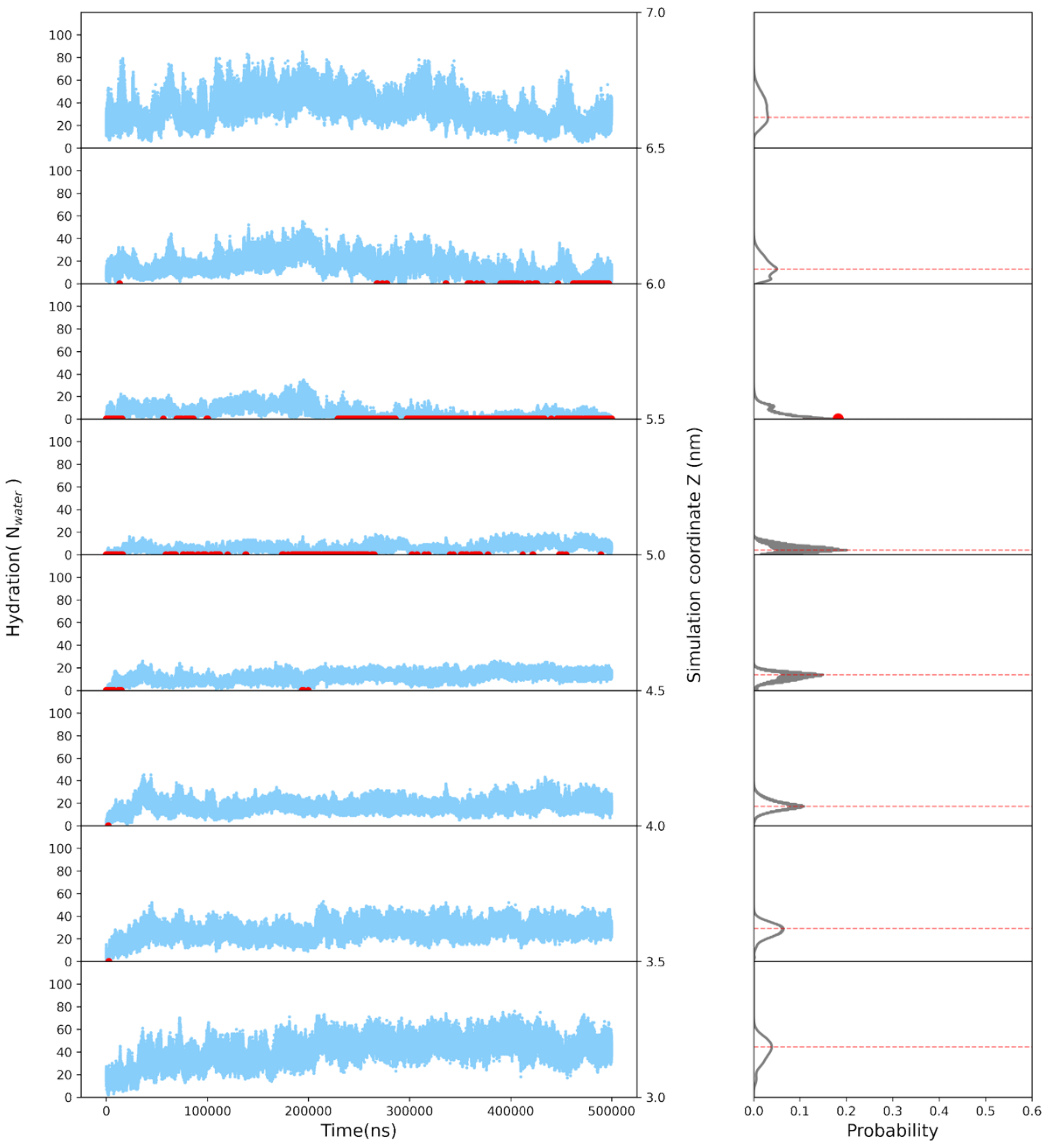

This Figure continues in next page

(l)

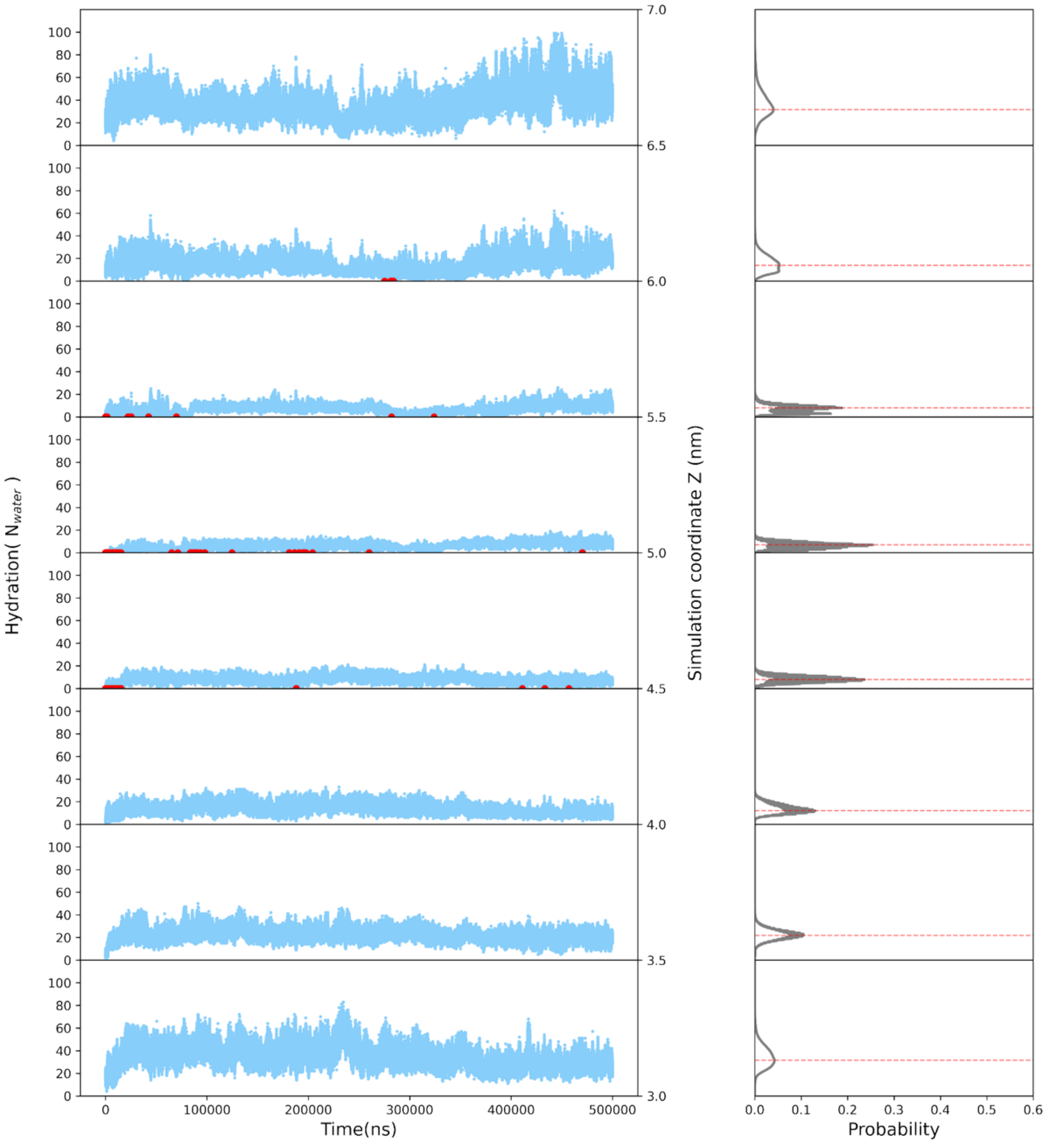

This Figure continues in next page

(G)

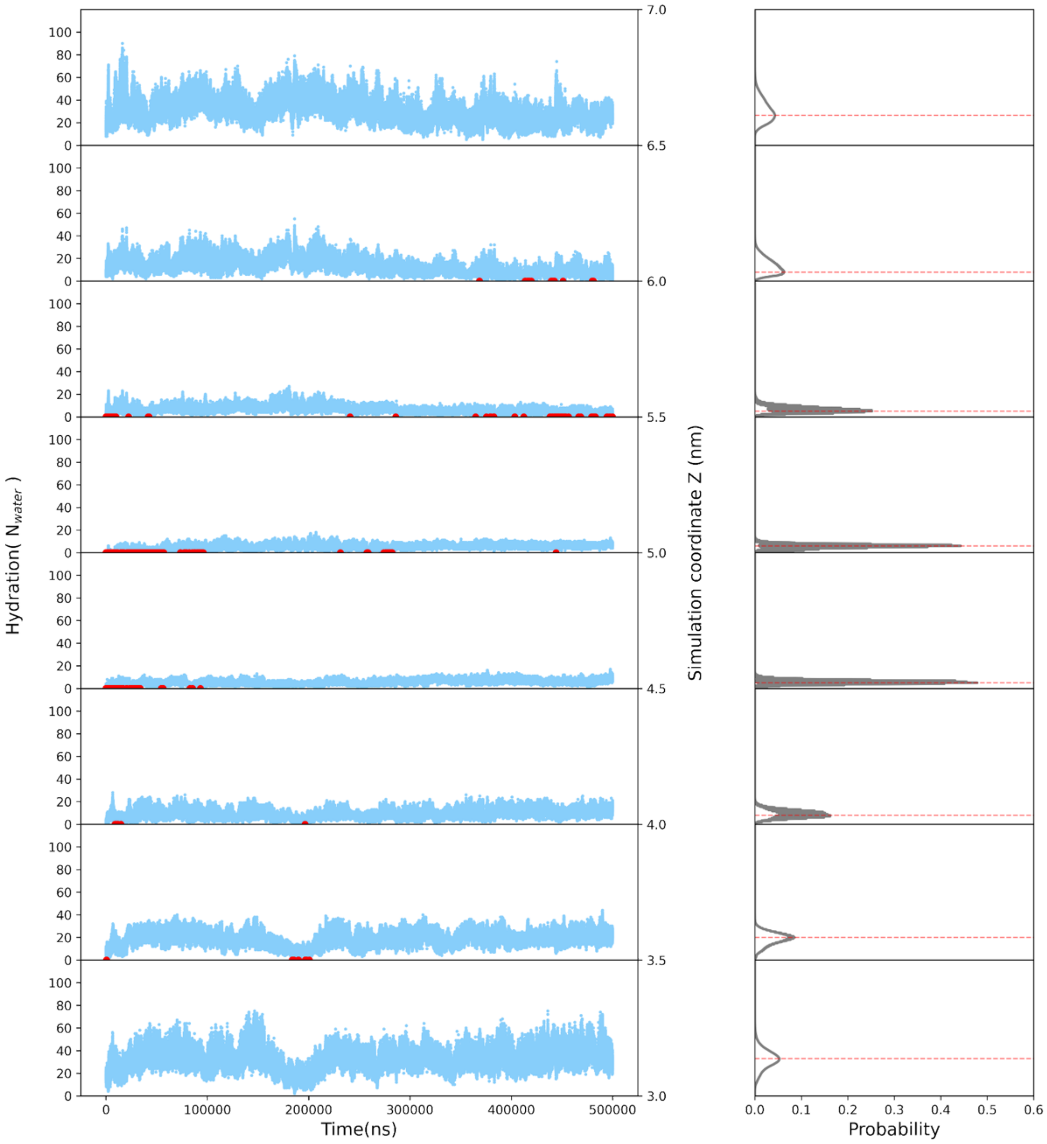

Figure S2. Water occupancy of the GerAB protein. (A)–(G) show data for runs 1 to 10. In each subplot, the left panel represents the time trace of water occupancy over the simulation, and the right panel represents the probability of water occupancy. Each plot is divided into eight zones on the vertical axis according to the Z coordinate of GerAB, and the number of water molecules is used as a measure of water occupancy. In the left panels, water occupancy is shown in blue, while zero occupancy is shown in red. In the right panels, the probability of water occupancy is shown in grey, with the highest probability indicated by red dotted lines, and zero occupancy in highest probability marked by a red dot.

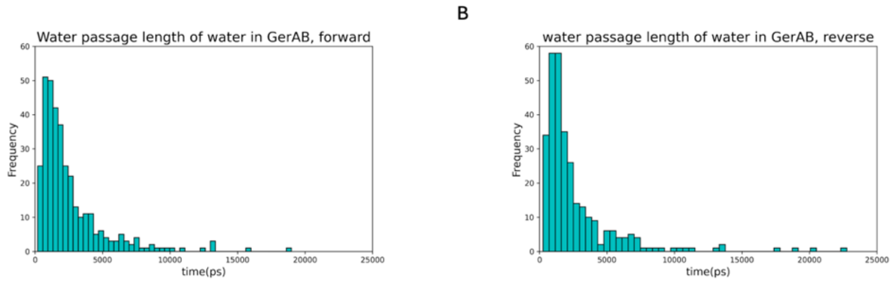

Figure S3. Distribution of the length of the water passage events.

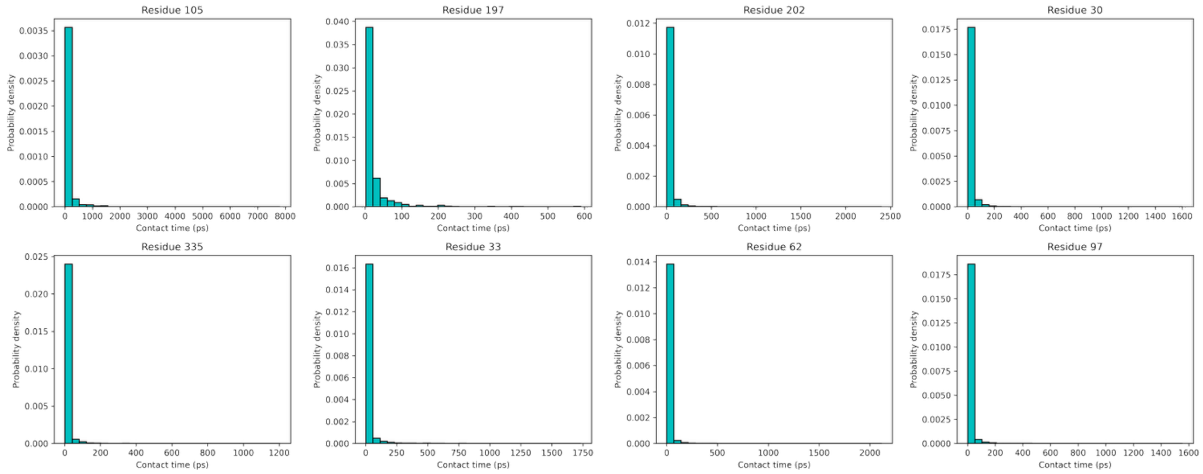

Figure S4. Residue–water contact time distribution. Each panel represents a high-contact residue, with probability density on the y-axis and contact time on the x-axis.

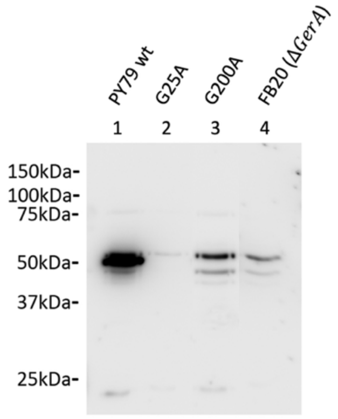

Figure S5. Western Blot of spores harbouring different gerAB variant against GerAA antibody. Both G25A and G200A spores exhibited similar bands as spore lacking *gerA* operon. Notably G25A mutants was reported to exhibit positive GerAA cross reaction material. Notably the G25A mutant strain tested here includes two copies of *gerA* operon. This figure shows partial Western Blot from one experiment; irrelevant samples were cropped for clarity.

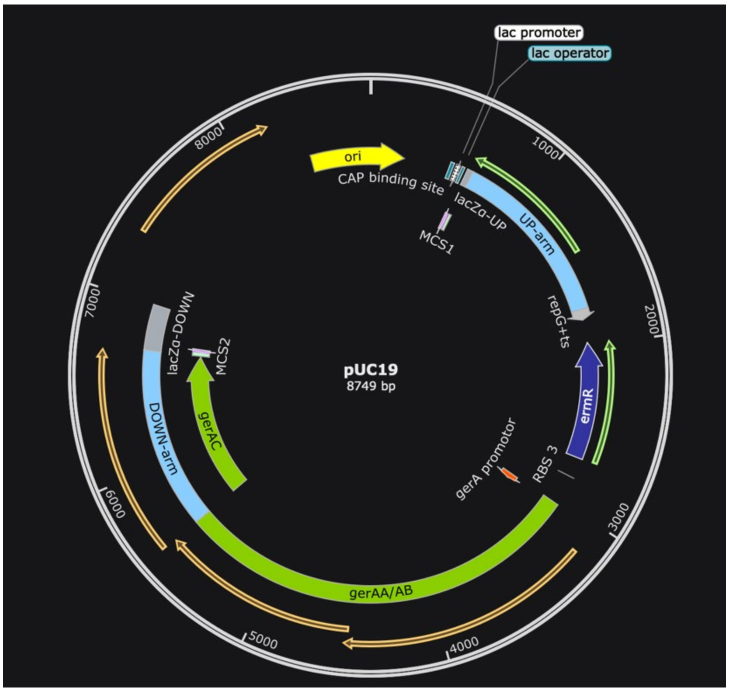

Figure S6. Gene construct for genome editing using a double-crossover strategy. The coding sequences of GerAA, GerAB, and GerAC are labelled in green, and the *gerA* operon promoter is labelled in orange. The upstream and downstream genomic homology arms are shown in light blue. The erythromycin resistance selection marker in *Bacillus subtilis* is labelled in dark blue.

```
GerRB|AAP07773.1      MNTMNKTKTVSPYFAFILLHSLQIGIGILGYQRVIL-KNAGYDAWISLIIAGIATHIVLF      59
GerKB|CAB12180.1      ----MEKARISIRQLFVMIIIFELGSSLLITPGSMA----GRDAWIAVLLGCAIGLFLFY      52
GerVB|ADE72615.1      ----MEKAKISASQLFILMVLFELGSSLLVPIAMDA----KQDAWLAILIGMVGSFVLFL      52
GerAB|KZD90227.1      MSQKQTPLKLNTFQGISIVANTMLGAGLLTLPRALTTKANTPDGWITLILEGFIFIFFIY      60
GerBB|CAB15598.1      --MRKSEHKLTFMQTLIMISSTLIGAGVLTLPRSAA-ETGSPSGWLMILLQGVIFIIIVL      57
                               :.    : ::    :* .:*             ..*: :::      ...

GerRB|AAP07773.1      CMLKMLEKD--GDLISIHTTTFGKWIGSIFSVIFTLYCLLFCLTVLRTYMEVIQVWIFPT      117
GerKB|CAB12180.1      LYQGIYQCYPNSSPKEYMDDMLGTKLSWLFSFLYILYFAYIAARVLRDFGEMLLTFAYHD      112
GerVB|ADE72615.1      VYHKLHSYYPDLLPTEYMQKIMGKGIGTVLAFVYILYFMYDAARVLRDFGAMLLTSAYPE      112
GerAB|KZD90227.1      LNTLIQKKHQYPSLFEYLKEGLGKWIGSIIGLLICGYFLGVASFETRAMAEMVKFFLLER      120
GerBB|CAB15598.1      LFLPFLQKNSGKTLFKLNSIVAGKFIGFLLNLYICLYFIGIVCFQARILGEVVGFFLLKN      117
                          : .        .      *. :. :: .    *         *    ::

GerRB|AAP07773.1      IKLWKLTLMFLLVTYYIIKGGFRSVTGICFWGIVL---PMFVVFFLVYPMKYAHFRNILP      174
GerKB|CAB12180.1      TPIIIVNALLMVVSIYAVRKGIEVLARAAELLFGAMYLLGAIGLVLIIVSGTIDPHNLKP      172
GerVB|ADE72615.1      TPLFIAHTLLMLVVIYTIRKGIEVVARSGEILFIFIYIFAIMGFVLIVCSGLIEFTNLQP      172
GerAB|KZD90227.1      TPIQVIILTFICCGIYLIVGGLSDVSRLFLFYLTV--TIIILLIVFGISFKIFDINNLRP      178
GerBB|CAB15598.1      TRMAVVVFIFLAVAIYHVGGGVYSIAKVYAYIFPI--TLIIFMMLLMFSFRLFQLDFIRP      175
                        :      ::    * :  *.  ::      :        . :.:       .   : *

GerRB|AAP07773.1      IFTHSPFDILVSAKQSALEFLGFETILI--FYPFIEKGKSLKRWAHAGIVFSTLIYVVLA      232
GerKB|CAB12180.1      VLANGISPVLHSVFTQTMYVPFGEVVLFVMIFPNLNDRKDVKKMGMIAMAISGLIVALTV      232
GerVB|ADE72615.1      VLEEGVLPVLKVAFTQTIYFPFAEAMVFTMILPYLKDQKKAKMTMLCATGLSGINLTITM      232
GerAB|KZD90227.1      VLGEGLGPIANSLTVVSISFLGMEVMLF--LPEHMKKKKYTFRYASLGFLIPIILYILTY      236
GerBB|CAB15598.1      VFEGGYQSFFSLFPKTLLYFSGFEIIFY--LVPFMRDPKQVKKAVALGIATSTLFYSITL      233
                      ::  .   .        : .   * :.   :   :.. *        .     :   :

GerRB|AAP07773.1      IVSFMYYSEGQLNHTIWPTLTMLKIIKVP--FIQRFEYIIIFVWFLIILPNLCLTIWSSC      290
GerKB|CAB12180.1      AINISVLDVDLTLRSQFPLLSTIQTIKVE-EFLDRLDVFFMLALIIGGFFKVSLYLYATV      291
GerVB|ADE72615.1      LINISVLGVDLTSRSQFPLLSTVQSIQVA-DFLERLDVFFMLALVIGGFIKISVLLYAAV      291
GerAB|KZD90227.1      IIVVGALTAPEVKTLIWPTISLFQSFELKGIFIERFESFLLVVWIIQFFTTFVIYGYFAA      296
GerBB|CAB15598.1      LIVIGCMTVAEAKTVTWPTISLIHALEVPGIFIERFDLFLQLTWTAQQFACMLGSFKGAH      293
                       : .            :* :: .: :::   *::*:: :: ..     :  .      :

GerRB|AAP07773.1      QTMKRSFHISFKFTLPFFI--FI---VFTASLFFKNRESINALNTVLSQAGLYIVY---A      342
GerKB|CAB12180.1      VGTSTLFKEKNPSQLAYPMGLGILILSITIATNFSEH-----LNEGLNVVPLYIHL----      342
GerVB|ADE72615.1      IGTANLFKIKSPSRLSYPLGFVILFMASTIASNFQEH-----LHEGLKVEMFILHM----      342
GerAB|KZD90227.1      NGLKKTFGLSTKTSMV----------IIGIAVFYFSL-----WPDDANQVMMYSDYLGYI      341
GerBB|CAB15598.1      IGLTEIFHLKNKNNAWLL-------TAMLAATFFITM-----YPKDLNDVFYYGTLLGYA      341
                            *  .                    :  :             .

GerRB|AAP07773.1      YIPILFLVHSLR-----WRFKNQSKKSSTDTP- 369
GerKB|CAB12180.1      --PFQLLFPLFLFIVAVWKKKRREKSKGEEAKK 373
GerVB|ADE72615.1      --PILAIIPSLLLLVAFLKNRKKQRG------- 366
GerAB|KZD90227.1      F-VSLFLLPFILFFIV-ALKRRITAK------- 365
GerBB|CAB15598.1      F-LIVITIPFFVWFLS-WIQKKIGRGQLQ---- 368
                             .  :         :.
```

Figure S7. Clustal Omega multiple sequence alignment of GerAB and homologs from *Bacillus* species, including GerRB from *Bacillus cereus*, and GerAB, GerBB, and GerKB from *Bacillus subtilis*, as well as GerVB from *Bacillus megaterium*. GenBank accession numbers are shown. High water contact residues identified in this study are highlighted, with conserved residues highlighted in blue, non-conserved residues highlighted in red.

Residue	*Mutation*	f_F/f_R	*atomF*	*atomR*
E202	**E202L**	0.0931/0.0891	OE1 44%; OE2 46%	OE1 49%; OE2 40%
I196	N/A	0.0290/0.0229	O 95%	O 96%
Y97	**Y97F**	0.0285/0.0247	OH 88%	OH 88%
N62	**N62L**	0.0264/0.0286	ND2 49%; OD1 48%	ND2 36%; OD1 60%
E105	**E105L**	0.0260/0.0388	OE1 56%; OE2 43%	OE1 53%; OE2 47%
T30	**T30V**	0.0258/0.0213	OG1 93%	OG1 94%
F198	N/A	0.0224/0.0166	O 97%	O 98%
S195	N/A	0.0208/0.0113	O 92%	O 88%
S197	**S197A**	0.0206/0.0172	O 65%; OG 34%	O 80%; OG 19%
S335	**S335A**	0.0201/0.0127	OG 56%; O36%	OG 55%; O 40%
F342	N/A	0.0195/0.0184	O 62%; CE2 13%	O 57%; CE2 17%
L345	N/A	0.0176/0.0130	O 88%	O 83%
T22	N/A	0.0156/0.0250	O 83%; OG1 17%	O 58%; OG1 41%
R33	**R33L**	0.0150/0.0135	NH2 39%; NE 37%; NH1 18%	NH2 35%; NE 41%; NH1 21%

Table S1. High contact frequency (f) residues identified in this study. Only both f_F and f_R higher than 0.01 were listed in this table, as shown in Figure 2 in main text. AtomF and AtomR listed the heavy atoms on each residue side chain that interact with the water oxygen in forward and reverse directions, respectively. O is backbone oxygen while OG, OE, OD, OH are sidechain oxygen. NH, NE, ND and CE are sidechain heavy atoms. The atom label follows that of a standard PDB file.

Mutant	Run 1	Run 2	Run 3	Run 4	Run 5
T30V	0	3	4	1	44
R33L	47	7	30	8	5
N62L	1	0	1	7	2
Y97F	1	4	54	151	2
E105L	1	1	0	0	24
S197A	56	0	3	30	4
E202L	2	1	1	2	0
S335A	1	0	0	18	1

Table S2. Water crossing passage numbers of mutant GerAB simulations in the reverse direction. Each mutant shows passage numbers per run.
